# Myo‐Guide: A Machine Learning‐Based Web Application for Neuromuscular Disease Diagnosis With MRI

**DOI:** 10.1002/jcsm.13815

**Published:** 2025-04-24

**Authors:** Jose Verdu‐Diaz, Carla Bolano‐Díaz, Alejandro Gonzalez‐Chamorro, Sam Fitzsimmons, Jodi Warman‐Chardon, Goknur Selen Kocak, Debora Mucida‐Alvim, Ian C. Smith, John Vissing, Nanna Scharff Poulsen, Sushan Luo, Cristina Domínguez‐González, Laura Bermejo‐Guerrero, David Gomez‐Andres, Javier Sotoca, Anna Pichiecchio, Silvia Nicolosi, Mauro Monforte, Claudia Brogna, Eugenio Mercuri, Jorge Alfredo Bevilacqua, Jorge Díaz‐Jara, Benjamín Pizarro‐Galleguillos, Peter Krkoska, Jorge Alonso‐Pérez, Montse Olivé, Erik H. Niks, Hermien E. Kan, James Lilleker, Mark Roberts, Bianca Buchignani, Jinhong Shin, Florence Esselin, Emmanuelle Le Bars, Anne Marie Childs, Edoardo Malfatti, Anna Sarkozy, Luke Perry, Sniya Sudhakar, Edmar Zanoteli, Filipe Tupinamba Di Pace, Emma Matthews, Shahram Attarian, David Bendahan, Matteo Garibaldi, Laura Fionda, Alicia Alonso‐Jiménez, Robert Carlier, Ali Asghar Okhovat, Shahriar Nafissi, Atchayaram Nalini, Seena Vengalil, Kieren Hollingsworth, Chiara Marini‐Bettolo, Volker Straub, Giorgio Tasca, Jaume Bacardit, Jordi Díaz‐Manera, the Myo‐Guide Consortium

**Affiliations:** ^1^ John Walton Muscular Dystrophy Research Centre Newcastle University Newcastle upon Tyne UK; ^2^ Department of Medicine (Neurology) The Ottawa Hospital Ottawa Canada; ^3^ Department of Genetics Children's Hospital of Eastern Ontario Ottawa Canada; ^4^ Ottawa Hospital Research Institute Ottawa Canada; ^5^ Copenhagen Neuromuscular Centre, Rigshospitalet Copenhagen University Hospital Copenhagen Denmark; ^6^ Department of Neurology, Huashan Hospital Fudan University Shanghai China; ^7^ Neuromuscular Disorders Unit, Neurology Department Hospital 12 de Octubre Madrid Spain; ^8^ Hospital Universitari Vall d'Hebron Barcelona Spain; ^9^ Neuromuscular Disorders Unit, Neurology Department Hospital Universitari Vall d'Hebron Barcelona Spain; ^10^ Department of Brain and Behavioural Sciences University of Pavia Pavia Italy; ^11^ Advanced Imaging and AI Center Mondino IRCCS Foundation Pavia Italy; ^12^ University of Pavia; Mondino IRCCS Foundation Pavia Italy; ^13^ UOC di Neurologia Fondazione Policlinico Universitario Agostino Gemelli IRCCS Rome Italy; ^14^ Fondazione Policlinico Universitario Agostino Gemelli Rome Italy; ^15^ Pediatric Neurology, Department of Woman and Child Health and Public Health, Child Health Area Università Cattolica del Sacro Cuore Rome Italy; ^16^ Hospital Clínico Universidad de Chile Santiago de Chile Chile; ^17^ Programa de Doctorado en Ciencias Médicas y Especialidad Escuela de Postgrado Facultad de Medicina Universidad de Chile Santiago Chile; ^18^ University Hospital Brno Brno Czech Republic; ^19^ Neuromuscular Disease Unit, Neurology Department Hospital Universitario Nuestra Señora de Candelaria Tenerife Spain; ^20^ Neuromuscular Disorders Unit, Department of Neurology Hospital de la Santa Creu i Sant Pau Barcelona Spain; ^21^ Biomedical Research Institute Sant Pau (IIB Sant Pau) Barcelona Spain; ^22^ Centro de Investigaciones Biomédicas en Red en Enfermedades Raras (CIBERER) Madrid Spain; ^23^ Department of Neurology Leiden University Medical Center Leiden The Netherlands; ^24^ C.J. Gorter MRI Center, Department of Radiology Leiden University Medical Center Leiden The Netherlands; ^25^ Northern Care Alliance NHS Foundation Trust Manchester UK; ^26^ Department of Translational Research and of New Surgical and Medical Technologies University of Pisa Pisa Italy; ^27^ Department of Neurology Pusan National University School of Medicine Busan Republic of Korea; ^28^ Centre de Référence des Maladies du Motoneurone, Department of Neurology Montpellier University Hospital Montpellier France; ^29^ Department of Neuroradiology, I2FH Platform Montpellier University Hospital Montpellier France; ^30^ Leeds Teaching Hospitals NHS Trust Leeds UK; ^31^ Paris Est University, APHP Henri‐Mondor University Hospital Créteil France; ^32^ Dubowitz Neuromuscular Centre UCL Great Ormond Street Institute of Child Health & Great Ormond Street Hospital London UK; ^33^ Department of Neuroradiology Great Ormond Street Hospital for Children NHS Foundation Trust London UK; ^34^ Department of Neurology Faculdade de Medicina da Universidade de São Paulo (FMUSP) São Paulo Brazil; ^35^ St George's University and St George's University Hospitals NHS Foundation Trust London UK; ^36^ Reference Center for Neuromuscular Disorders CHU La Timone, Aix‐Marseille University Marseille France; ^37^ Aix‐Marseille University, CRMBM, CNRS UMR 7339 Marseille France; ^38^ Department of Neuroscience, Mental Health and Sensory Organs (NESMOS) SAPIENZA University of Rome Rome Italy; ^39^ Neuromuscular and Rare Disease Centre, Neurology Unit, Sant'Andrea Hospital Rome Italy; ^40^ Neuromuscular Reference Center, Department of Neurology, Universitair Ziekenhuis van Antwerpen Universiteit Antwerpen Antwerp Belgium; ^41^ University Hospital Raymond‐Poincaré Garches France; ^42^ Neurology Department, Shariati Hospital, Neuromuscular Research Center Tehran University of Medical Sciences Tehran Iran; ^43^ National Institute of Mental Health and Neurosciences (NIMHANS) Bengaluru India; ^44^ Translational and Clinical Research Institute Newcastle University Newcastle upon Tyne UK; ^45^ Interdisciplinary Computing and Complex BioSystems (ICOS) Research Group, School of Computing Newcastle University Newcastle upon Tyne UK

**Keywords:** artificial intelligence, differential diagnosis, machine learning, MRI, neuromuscular diseases

## Abstract

**Background:**

Neuromuscular diseases (NMDs) are rare disorders characterized by progressive muscle fibre loss, leading to replacement by fibrotic and fatty tissue, muscle weakness and disability. Early diagnosis is critical for therapeutic decisions, care planning and genetic counselling. Muscle magnetic resonance imaging (MRI) has emerged as a valuable diagnostic tool by identifying characteristic patterns of muscle involvement. However, the increasing complexity of these patterns complicates their interpretation, limiting their clinical utility. Additionally, multi‐study data aggregation introduces heterogeneity challenges. This study presents a novel multi‐study harmonization pipeline for muscle MRI and an AI‐driven diagnostic tool to assist clinicians in identifying disease‐specific muscle involvement patterns.

**Methods:**

We developed a preprocessing pipeline to standardize MRI fat content across datasets, minimizing source bias. An ensemble of XGBoost models was trained to classify patients based on intramuscular fat replacement, age at MRI and sex. The SHapley Additive exPlanations (SHAP) framework was adapted to analyse model predictions and identify disease‐specific muscle involvement patterns. To address class imbalance, training and evaluation were conducted using class‐balanced metrics. The model's performance was compared against four expert clinicians using 14 previously unseen MRI scans.

**Results:**

Using our harmonization approach, we curated a dataset of 2961 MRI samples from genetically confirmed cases of 20 paediatric and adult NMDs. The model achieved a balanced accuracy of 64.8% ± 3.4%, with a weighted top‐3 accuracy of 84.7% ± 1.8% and top‐5 accuracy of 90.2% ± 2.4%. It also identified key features relevant for differential diagnosis, aiding clinical decision‐making. Compared to four expert clinicians, the model obtained the highest top‐3 accuracy (75.0% ± 4.8%). The diagnostic tool has been implemented as a free web platform, providing global access to the medical community.

**Conclusions:**

The application of AI in muscle MRI for NMD diagnosis remains underexplored due to data scarcity. This study introduces a framework for dataset harmonization, enabling advanced computational techniques. Our findings demonstrate the potential of AI‐based approaches to enhance differential diagnosis by identifying disease‐specific muscle involvement patterns. The developed tool surpasses expert performance in diagnostic ranking and is accessible to clinicians worldwide via the Myo‐Guide online platform.

## 1 Introduction

Neuromuscular diseases (NMDs) are a heterogeneous group of disorders affecting the function of skeletal muscle, often characterized by progressive muscle wasting and its substitution by non‐contractile fatty and connective tissue. NMDs can manifest across all age groups and are associated with a wide range of clinical presentations, from mild muscle weakness to severe disability, and are sometimes associated with life‐threatening complications [1].

An early and accurate diagnosis is a critical step in effectively managing and treating NMDs. However, diagnosing these disorders presents a challenge due to their diverse and overlapping clinical presentations. Magnetic resonance imaging (MRI) has increased in popularity during the last decades as a tool for studying NMDs, as it allows for muscle and fatty tissue differentiation [2, 3].

Different methods exist to assess fat replacement, which can be categorized into two broad groups: quantitative and semi‐quantitative. The main quantitative technique for intramuscular fat assessment is the chemical shift‐based water and fat separation (or Dixon) technique. This imaging technique separates fat and water signals in different images, allowing the reconstruction of a fat fraction (FF) map [4]. A precise measure of the FF can then be retrieved by selecting regions of interest (ROIs) in the FF map [5]. Although this technique yields a reliable measurement, most of the available retrospective data consist of qualitative T1w scans [2]. The main semi‐quantitative techniques are based on the Mercuri score, a visual 5‐point scale ranging from 0 (*healthy muscle*) to 4 (*completely fat‐replaced muscle*) [6].

Muscle fatty replacement is not random, and different disease‐causing genes have been linked to the selective involvement of groups of muscles. This has allowed the identification of the so‐called patterns of fat replacement, which are useful in proposing a potential diagnosis [7, 8, 9]. However, the number of patterns described has increased considerably, making the identification of patterns a complex and time‐consuming task that requires a high degree of expertise. Moreover, many of these patterns are overlapping, and there is no atlas collecting and keeping findings up to date [9]. This complexity is ideally suited for the capability of artificial intelligence (AI) techniques, allowing for a deeper analysis of muscle fat replacement patterns [10].

Emerging AI tools have proven to be useful for the diagnosis of NMDs using MRI data [11, 12, 13]. However, current studies are focused on a relatively small number of NMDs, and the identified patterns lose specificity when compared against a larger pool of disorders [14]. In 2020, we published a paper sharing the results of a pilot machine learning–based (ML) tool for diagnosing multiple NMDs using MRI data [15]. We demonstrated that supervised ML techniques are useful for NMD diagnosis, and we also identified several limitations that require addressing:

- AI models are often considered hard‐to‐interpret black boxes. However, in clinical settings, model explainability is critical to validate the results of such models. Furthermore, understanding the decision process of a diagnosis model can help identify novel insights about the disease's evolution. Multiple AI explainability techniques have been presented; however, these are yet to be used and adapted for understanding muscle involvement in NMD patients.
- The impact of AI models for patient diagnostics is limited by their accessibility, and clinicians require an interface to interact with such models.
- Current approaches have targeted a limited number of diseases, and there is a need for a general model, able to generalize over a larger pool of disorders.

**FIGURE 1:**
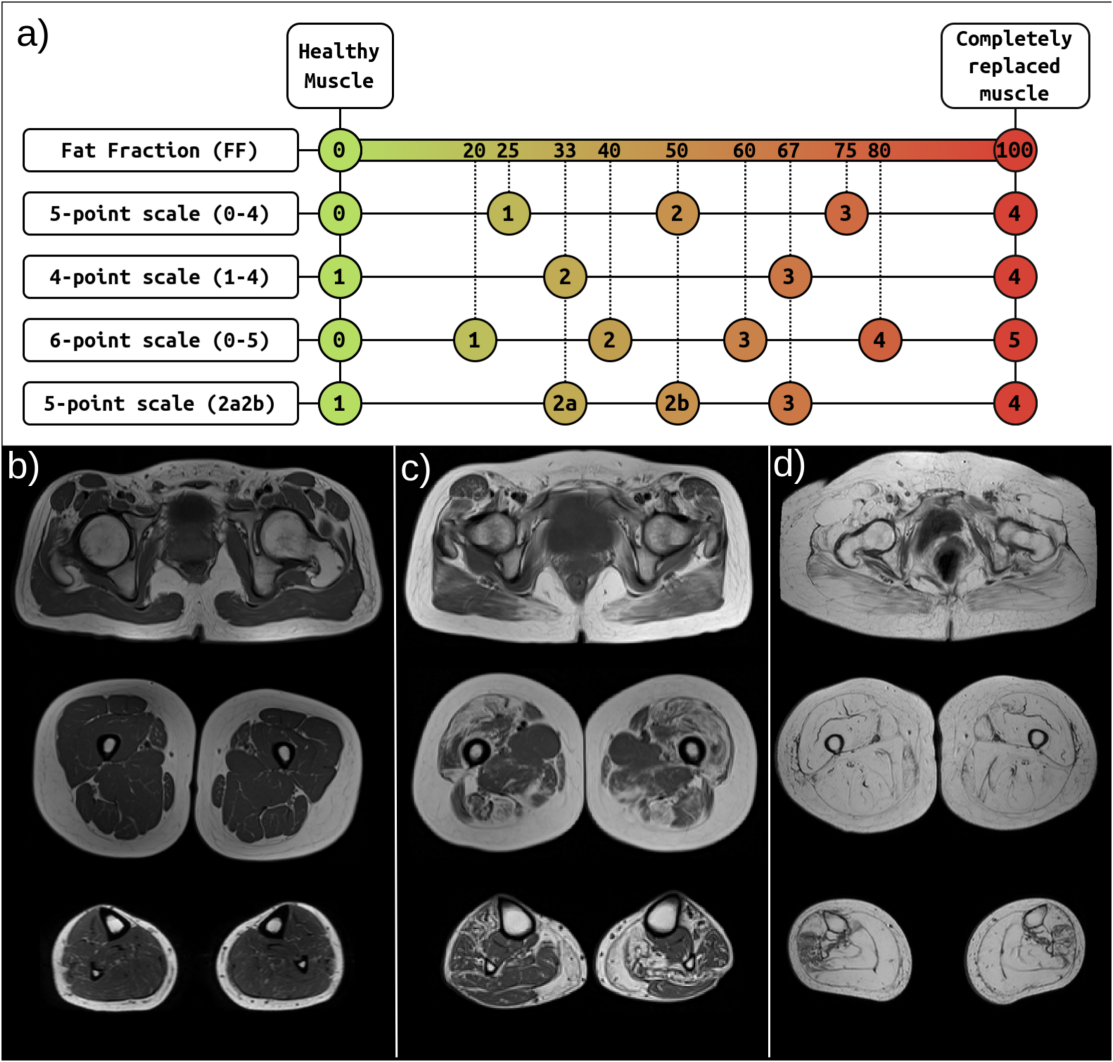
(a) Representation of the different muscle fat scales found in the dataset. Each row represents a different scale, and each coloured circle represents a possible value. The fat fraction scale is continuous, and the rest are discrete. Discrete scales are projected over the FF scale to show differences in muscle fat quantisation. (b) MRI examples of healthy muscle, (c) intermediate‐stage muscle and (d) late‐stage muscle. Fatty tissue appears with high intensity in the MRIs (white/light grey), whereas muscle tissue appears with low intensity (dark grey). The top row of the MRIs represents a pelvis/waist‐level axial slice, the second row represents a thigh axial slice, and the last row represents a calf axial slice.

Additionally, collecting a dataset eligible for AI analysis demands joining retrospective data from multiple sites and multiple studies, leading to different types of heterogeneity in the data [16]:

- Scale: Intramuscular fat replacement can be measured using multiple continuous and discrete scales. All scales describe a value for healthy and completely fat‐replaced muscle; however, discrete scales do not align on how the severity is quantized. Unless the proportion of samples using each scale is equal across NMDs, the degree of muscle involvement may be emphasized more in some conditions than others in the compiled dataset. This effect, known as scale information leakage, may lead to false predictors in diagnosis (Figure 1a).
- Muscle grouping: Researchers often group muscles due to similar function or involvement, spatial proximity or low MRI scan resolution (i.e. grouping the psoas and iliacus muscles into iliopsoas). Integrating data from studies using different feature groups requires a flexible data model able to unify the feature space.
- Bilateral/unilateral scores: Muscle fat replacement can be scored for both sides of the body (left and right) at the expense of increasing the time required for scoring. However, most NMDs show symmetrical muscle involvement, resulting in redundant information when reporting the data. For the sake of time and visual clarity, some researchers choose to report unilateral or average scores.

This heterogeneity can bias classification models if not accounted for. Therefore, a data harmonization pipeline is required to adapt muscle fat replacement measurements to a unique scale.

In this paper, we aim to provide solutions to these limitations. We present the following:

- A software pipeline for multi‐study and/or multicentre muscle MRI data harmonization.
- The collection of the largest available multi‐study dataset for NMDs, consisting of 3463 patient records across 40 diseases.
- A scalable NMD diagnosis ensemble model based on the XGBoost algorithm.
- An adaptation of current AI explainability methods (SHapley Additive exPlanations [SHAP]) and the creation of novel visualization techniques to identify and understand discriminative patterns of muscle involvement in NMDs.
- Myo‐Guide, a web portal hosting the diagnosis model and serving as a platform for pushing forward the role of MRI in the study of NMDs.

## 2 Materials and Methods

### 2.1 Study Data

We established the Myo‐Guide consortium to collect a large MRI data repository on a wide range of NMDs. Thirty‐four clinical sites from Europe, America and Asia collaborate by sharing MRI scans and muscle fat data via the secure Myo‐Share platform [17]. Published datasets of muscle fat replacement were also incorporated [S1–S24]. Age and sex were collected when available.

All included patients required a confirmed genetic diagnosis. T1‐weighted lower limb MRI scans were collected, and all muscles were scored using a 5‐point scale (values from 0 to 4) by an experienced clinician. Left and right muscles were scored to assess asymmetric involvement. Already existing muscle fat scores were directly integrated. The number of samples extracted from the literature are as follows: GNE (31), SarcoG (155), PABPN1 (277), FKRP (93), DUX4 (153), LMNA (37), CAPN3 (82), SMN1 (55), OPDM (77), ANO5 (26), DMD (119), DMPK (134) and PYGM (46). All samples of the disorders not mentioned were manually scored by Diaz‐Manera J., Bolaño‐Diaz C. and Selen Kocak G.

For longitudinal data, each time point was considered a new sample. To avoid data leakage from non‐independent samples, we grouped samples by patient and used each group exclusively for training or testing.

As of submission, the Myo‐Guide Consortium has gathered 3463 lower limb muscle MRI samples from patients genetically diagnosed with 40 NMDs. We selected all diseases with at least 30 samples, resulting in 2961 samples from 20 NMDs. An overview of this subset and labels used for each disease is available in Table 1. The labels represent either the main gene causing each disease or a short disease name. Please note that the DMD class (dystrophinopathies) includes both Duchenne and Becker muscular dystrophies.

**TABLE 1:** Data overview.

Disease	Label	*N*	Mean Age	Sex		Side			Scale				
				M	F	B	A	U	0–4	0–5	1–4	2a2b	FF
Anoctaminopathy	ANO5	116	50.06	73	41	116	0	0	90	0	26	0	0
Calpainopathy	CAPN3	139	36.16	61	74	76	18	45	54	0	66	0	19
Myotonia congenita	CLCN1	31	46.07	16	15	31	0	0	31	0	0	0	0
Dystrophinopathies	DMD	179	27.22	179	0	157	0	22	106	22	0	51	0
MD type 1	DMPK	142	45.65	84	53	142	0	0	142	0	0	0	0
FSHD type 1	DUX4	489	46.34	145	143	349	140	0	336	13	0	0	140
Dysferlinopathy	DYSF	594	38.48	284	298	238	356	0	238	0	0	0	356
LGMDR9	FKRP	112	38.17	32	44	19	46	47	55	11	0	0	46
Pompe disease	GAA	116	43.08	28	31	116	0	0	116	0	0	0	0
GNE myopathy	GNE	75	34.27	32	38	57	0	18	75	0	0	0	0
Hypokalaemic periodic paralysis	HypoPP	41	45.22	27	14	41	0	0	41	0	0	0	0
Laminopathies	LMNA	85	37.88	21	23	18	67	0	77	0	8	0	0
OPDM	OPDM	77	52.21	37	40	0	69	8	77	0	0	0	0
OPMD	PABPN1	288	62.84	130	112	184	104	0	238	0	8	0	42
McArdle disease	PYGM	67	43.76	34	32	21	46	0	21	0	0	0	46
Hyperkalaemic periodic paralysis	SCN4A	30	43.87	16	14	30	0	0	30	0	0	0	0
Spinal muscular atrophy	SMN1	71	19.24	27	16	16	0	55	16	0	0	55	0
Sarcoglycanopathies	SarcoG	170	20.98	55	51	84	64	22	84	22	64	0	0
Titinopathy	TTN	81	18.38	41	36	81	0	0	32	49	0	0	0
VCP‐related myopathy	VCP	58	51.64	31	12	4	0	54	4	0	54	0	0

*Note:* Each disease has a shorter label assigned, which has been used throughout this document. The age column shows the mean value, whereas the rest shows the sample counts. Counts of samples with an unknown sex are not represented. Sex is coded as M (male) and F (female). The scores side is encoded as B (bilateral score), A (average scores) and U (unilateral scores). The different scales used to measure fat replacement are the 5‐point Mercuri score (0–4), 6‐point Mercuri score (0–5), 4‐point Mercuri score (1–4), 5‐point Mercuri score with two subcategories on point 2 (2a2b) and Dixon fat fraction (FF). The definition of each scale is available in Figure 1a.

Abbreviations: FSHD, facioscapulohumeral muscular dystrophy; MD, myotonic dystrophy; OPDM, oculopharyngodistal myopathy; OPMD, oculopharyngeal muscular dystrophy.

### 2.2 Muscle Score Harmonization

To merge existing datasets with different muscle groupings, we defined two simple operations: muscle merging and group expansion. Muscle merging joins multiple muscles into a single muscle group by averaging the scores of each individual muscle (*peroneus brevis* and *peroneus longus* into *peronei*). Group expansion divides a muscle group into its individual muscles by assigning the same score to each muscle (*biceps femoris* into *biceps femoris long head* and *biceps femoris short head*).

We averaged bilateral scores into mean muscle scores. The asymmetry of each muscle was defined as the difference between both sides, and the mean and standard deviation of the asymmetry were calculated for each patient.

We normalized all scales to a range between 0 (*healthy muscle*) and 100 (*completely fat‐replaced muscle*), considering the range of values of each original scale. Fat fraction scores were not normalized, as they already range between 0 and 100. Then, we rescaled the scores by subtracting the leave‐one‐out mean of each patient from each muscle fat replacement feature. This transformation removes the information representing the overall amount fatty replacement, which indicates the disease stage of the patient. To keep this crucial information, we added the mean muscle involvement of each patient (before scaling the scores into relative scores) as an additional feature. After processing, fat score features are left in a range between −100 and 100. Negative values indicate muscles with a lower fat content than the average of the patient, and vice versa.

### 2.3 Missing Data

The final dataset consists of an aggregate of patient data from various sources and studies, collected following distinct protocols. The muscles reported by different sources vary, leading to blocks of missing information for some disorders. We imputed missing values using the K‐nearest neighbour (KNN) algorithm, which has proven to perform well with blocks of missing data, as it does not make strong assumptions about the distribution of the data, leverages information from complete features and captures multivariate relationships [S25, S26].

### 2.4 Oversampling and Data Augmentation

To mitigate the impact of class imbalance, the Synthetic Minority Oversampling Technique for Nominal and Continuous (SMOTE‐NC) was employed [S27]. This technique involves generating synthetic samples for the minority classes, thereby increasing their representation in the dataset. SMOTE‐NC helps prevent the model from being biased towards the majority class, resulting in more balanced training data.

Additionally, SMOTE‐NC allows to further mitigate scale information leakage by blurring the quantization after rescaling the fat scores. We modified SMOTE‐NC to oversample all classes, including the majority class, regulated by the parameter *augment_factor*. Resampling was performed only on the training subset to avoid leaking training information into the validation data. Hence, we use SMOTE‐NC as a general data augmentation technique.

### 2.5 Model Training and Evaluation

The processed data were used to train and validate an XGBoost classifier using a nested cross‐validation approach. The outer cross‐validation splits the data into 10 stratified folds to provide an unbiased estimate of the model's performance. Samples were grouped by patient to avoid mixing multiple samples of the same patient in different folds. The inner cross‐validator splits the data into 5 stratified folds to optimize the hyperparameters.

We conducted a comparative analysis of the performance of XGBoost and random forest, the latter being another widely used decision tree‐based algorithm, which we employed in our previous pilot study [18] Additionally, we evaluated the efficacy of KNN for the classification task [19], considering that KNN was also utilized for data imputation in this study.

Given the presence of class imbalance in the dataset, the optimization was based on the balanced accuracy metric, which accounts for the unequal distribution of classes and prevents bias towards the majority class. This approach aimed to enhance the model's generalizability and its ability to accurately classify instances from all classes, including the minority ones. Balanced accuracy is defined as the average recall obtained in each class. We also calculated a class‐weighted top‐n accuracy, $W_{n}$, defined as in Equation (1).

(1) $$W_{n}=\frac{\sum_{i=1}^{S}w_{y_{i}}\cdot I\left(y_{i}\in P_{n,i}\right)}{\sum_{i=1}^{S}w_{y_{i}}}$$

where $w_{y_{i}}$ is the weight assigned to the class y of the sample i, S is the total number of samples, and I is the indicator function that returns 1 if the true label $y_{i}$ is within the top n predictions $P_{n,i}$ and 0 otherwise. We calculate the class weights as the inverse of the class frequencies, expressed in Equation (2).

(2) $$w_{i}=\frac{S}{\text{count}\left(y_{i}\right)}$$

where $\text{count}\left(y_{i}\right)$ is the number of samples belonging to the class $y_{i}$.

We also evaluated the model by calculating the One‐vs‐Rest and One‐vs‐All receiver operating curves (ROCs) and precision–recall curves (PRCs). To calculate the One‐vs‐Rest PRCs, the multiclass classification is decomposed into multiple binary classification problems. For each class, we calculated the ROC by treating that class as the positive class and all others combined as the negative class. This allows us to assess classifier performance for each class against the rest.

When calculating the One‐vs‐One PRCs, the results are again broken down into multiple binary classification problems. For each pair of diseases, the model is evaluated twice: first by considering one of the diseases as the positive class and the other as the negative class and then the other way around. The results of both are averaged to represent the PRC curve for the pair of diseases.

### 2.6 Hyperparameter Optimization

We optimized the hyperparameters using Bayesian optimization techniques. Bayesian optimization works by sampling points in the parameter space using the results of previous sampled points, converging orders of magnitude faster than a grid search of parameters and yielding better results than a random parameter search [S28].

A tree‐structured Parzen estimator (TPE) was used to sample points in the parameter space [S29], and each parameter combination was tested using the inner cross‐validator. Hyperparameter optimization was performed using the Optuna framework [S30].

The TPE sampler was initialized with 30 points, and 60 different hyperparameter combinations were sampled at each fold. The parameter combinations with performance over the 90th percentile of each outer cross‐validation fold were used to create an ensemble of XGBoost models by averaging the outputs of each individual model. Given that the SHAP values used for explaining the model are calculated using the raw marginal outputs of the XGBoost models, we averaged the marginal outputs of the ensemble and applied the softmax function to the result, defined as in Equation (3).

(3) $$\text{soft}\max\left(x\right)=\frac{\;e^{x_{\mathrm{ik}}-\max\left(X_{i}\right)}}{\sum_{k=1}^{K}e^{x_{\mathrm{ik}}-\max\left(X_{i}\right)}}$$

where $X_{i}$ represents ith row of the input array X, corresponding to the set of scores for the ith instance. $x_{\mathrm{ij}}$ is the score for the jth class in the ith instance, and K is the total number of classes.

### 2.7 AI Versus Clinical Experts' Comparison

We compared the model's performance to that of field experts using a dataset composed entirely of previously unseen mMRI scans. For each neuromuscular disorder, one scan was randomly selected. However, due to the unavailability of new mMRI scans for every disorder, this set contained 14 scans representing 14 different NMDs: FKRP, DMD, VCP, DMPK, GNE, CAPN3, SCN4A, ANO5, SarcoG, DYSF, DUX4, PABPN1, LMNA and GAA.

Four clinicians with long expertise in the use of MRI for diagnosis of patients in the clinical setting participated in this experiment (C.B., G.T., V.S. and J.D.). They were blinded to the diagnosis for each scan and were told that NMDs could repeat within the test dataset. Some mMRIs included muscles not used in the model (paraspinal, arm muscles, etc.), which were manually masked out of the scans to make a fair comparison. Age and sex data were available for all patients except for patient P14, for whom the age at scan was not recorded. Most MRI scans covered the pelvis, thigh and lower leg muscles; however, patients P1 and P12 did not have lower leg images, and patients P8, P11 and P13 were missing some upper pelvic muscles.

The experts were asked to select, in order of preference, 3 potential diagnoses. They were allowed to look at the mMRI scan and use any other visual features, such as muscle morphology, intramuscular fat distribution and texture. Some experts were unable to provide the full raking for some patients due to uncertainty. These missing values were imputed by selecting a random diagnosis.

Muscles were manually scored by C. Bolaño‐Diaz and processed following the pipeline defined in this manuscript. The model was used to produce predictions, and their accuracy was evaluated using top‐K accuracy curves. SHAP values were used to explain each prediction, and a round table between the experts was held to evaluate the results of the experiment and gain insights about the patterns identified by the model.

## 3 Results

### 3.1 Harmonization and Preprocessing

**FIGURE 2:**
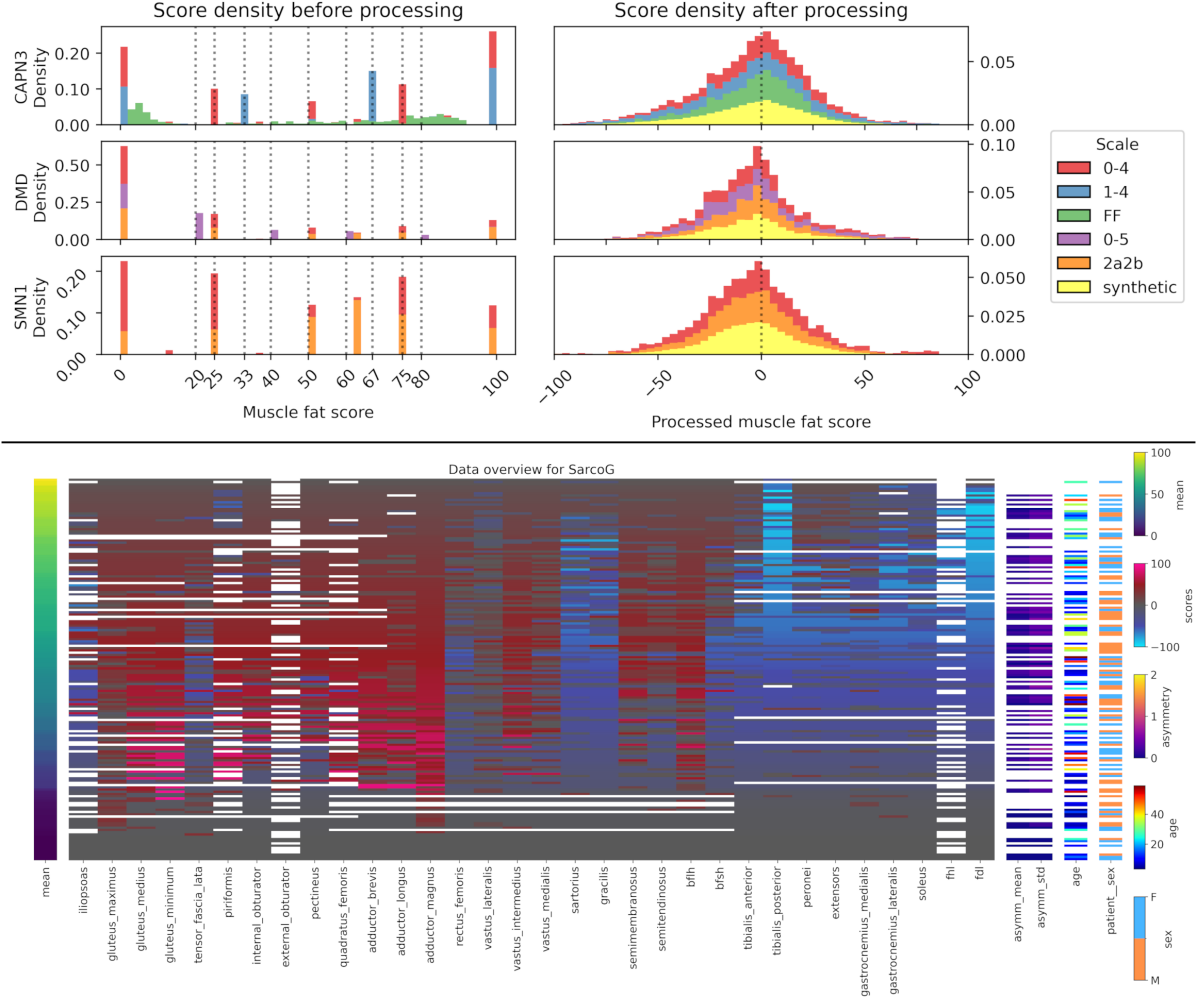
Distribution of muscle fat scores before (a) and after (b) processing for CAPN3, DMD and SMN1. Normalized stacked densities are shown for each different scale. The discrete values shown in Figure 1a are highlighted in the left figure. Scores outside of the discretised values correspond to mean values from the left and right legs. (c) Heatmap of the data for SarcoG. Patient samples are shown in rows and features in columns. Rows are sorted by mean fat score, with late‐stage patients in the upper rows and early‐stage patients in the lower rows. Asymmetry is calculated as the difference between each left and right muscle, and the mean and standard deviation of the asymmetry of all muscles are added as features for each patient. Empty (white) spaces represent missing data. Muscle abbreviations: biceps femoris long head (bflh), biceps femoris short head (bfsh), flexor hallucis longus (fhl) and flexor digitorum longus (fdl). The extensor digitorum longus and extensor hallucis longus have been grouped and named ‘extensors’.

Our proposed harmonization and preprocessing pipeline scales all intramuscular fat scores to a range from −100 to 100. In this new space, negative values indicate muscles less affected than the average of the patient and vice versa. In Figure 2a,b, we show the effect of this pipeline: Before processing, scale information can be easily recovered from the muscle fat scores. Given that the distribution of scales is not equal across diseases, this information would introduce bias in the model if not accounted for. After processing, scores show a similar distribution independently of the scale and do not leak scale information into the data. A complete comparison across all target diseases can be found in Figure S1.

Scaling the fat scores by subtracting the leave‐one‐out mean helps in highlighting the relative involvement of muscles independently of the disease stage, allowing the identification of patterns of muscle involvement more effectively than by plotting absolute fat scores. Figure 2c shows an example of the processed data for the SarcoG class as a heatmap sorted by mean muscle fat score. We can observe an early relative involvement of the pelvic muscles (glutei, piriformis, obturators, pectineus, quadratus femoris and adductors) except for the iliopsoas and tensor fasciae latae. We can also observe a relatively low involvement of the lower leg muscles (tibialis anterior and posterior, peronei, gastrocnemii, soleus and flexors) at an intermediate disease stage. The tibialis posterior and flexor digitorum longus muscles show a minimum relative involvement at a late stage of the disease. Heatmaps for the rest of the target diseases can be found in Figures S2–S20.

### 3.2 Diagnosis Model

The degree of intramuscular fat replacement, age, sex and asymmetry were used as input features to train an XGBoost classifier ensemble. The model ensemble obtained an average balanced accuracy of 64.8% ± 3.4%. We also assessed the performance of the model in suggesting multiple diagnoses by measuring the weighted top‐N accuracy. This metric evaluates the percentage of samples where the ground truth is within the top‐N choices of the model, weighting each class to avoid the effect of class imbalance. We obtained a weighted top‐3 accuracy of 84.7% ± 1.8% and a weighted top‐5 accuracy of 90.2% ± 2.4%.

**FIGURE 3:**
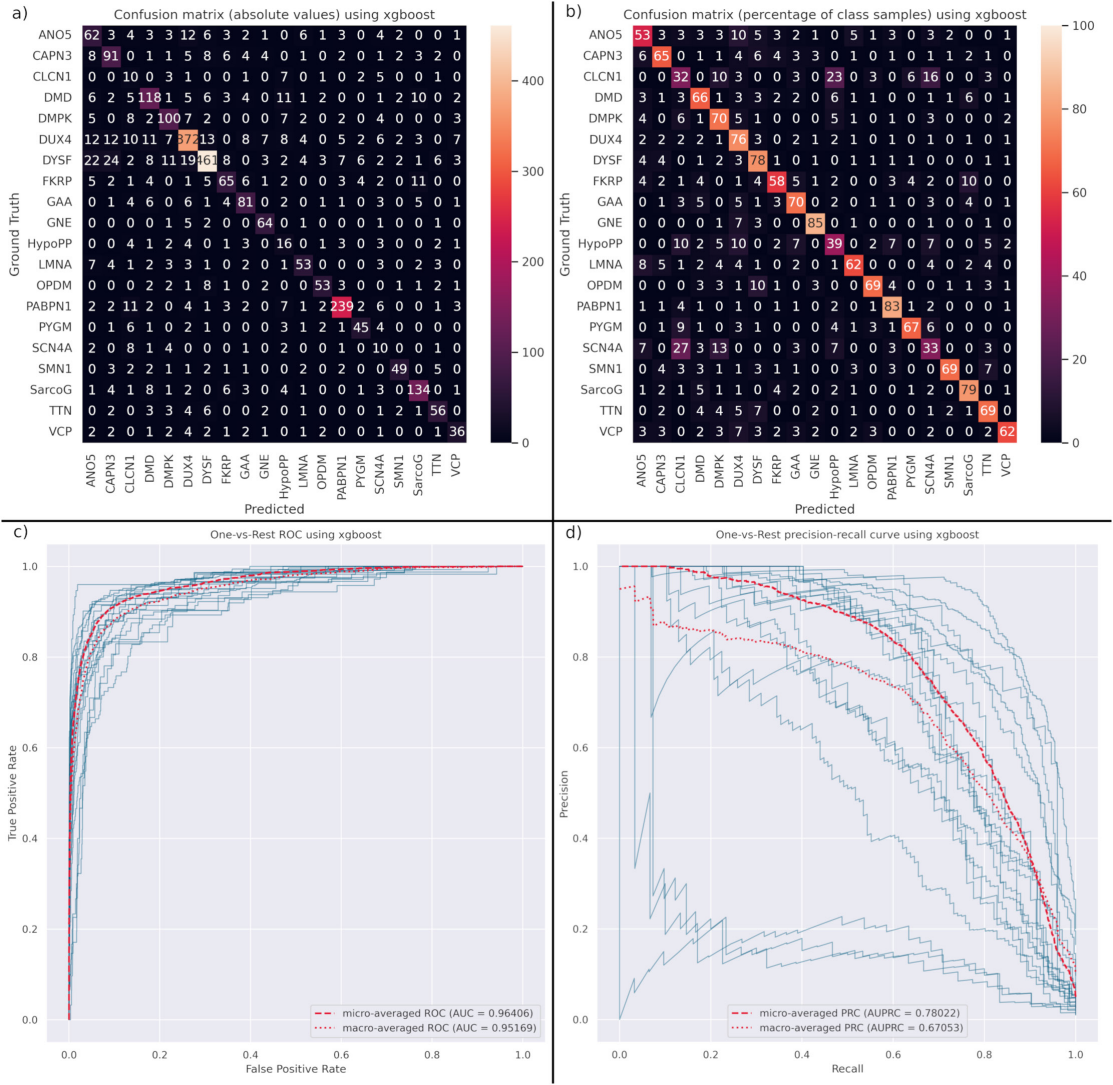
Evaluation plots for the XGBoost model ensemble. (a) Confusion matrix of the model with the test data. The ground truth is shown in rows and the model predictions are in columns. The diagonal corresponds to the correctly predicted samples. (b) Confusion matrix normalized by ground truth (rows). (c) One‐vs‐Rest receiving operating curves for each disease (blue) and micro‐ and macro‐averaged curves (red). The area under the curve for the micro‐ and macro‐averaged curves is shown in the legend. (d) One‐vs‐Rest precision–recall curves for each disease (blue) and micro‐ and macro‐averaged curves (red). The area under the precision–recall curve for the micro‐ and macro‐averaged curves is shown in the legend.

We complemented these metrics by calculating the confusion matrix, ROC curves and PRCs of the model ensemble (Figure 3). The confusion matrix (Figure 3a) breaks down the results of the ensemble predictions by class, comparing the model predictions against the ground truth. To compensate for class imbalance and make the confusion matrix easier to interpret, we normalized it by ground truth (Figure 3b). The model ensemble predicted correctly 80% or more of the patients labelled with GNE and PABPN1 and between 70% and 80% of the patients labelled with DMPK, DUX4, DYSF, GAA and SarcoG. However, it struggled in predicting patients labelled with ANO5 and FKRP, guessing correctly in 53% and 58% of the patients respectively. Finally, we consider that the model failed in predicting CLCN1, HypoPP and SCN4A, guessing correctly under 40% of the patients.

The latter three diseases are the least represented in the dataset (31, 41 and 30 samples respectively), and they are characterized by a mild or absent fatty replacement in muscle, except for some patients (Figure S21) [20]. This suggests that the lack of muscle involvement lowers the performance of the model in predicting these diseases, requiring additional features for providing a precise diagnosis.

The ROCs (Figure 3c) seem to offer an overoptimistic measurement of the model performance with an AUC over 0.95 for both the micro‐ and macro‐averaged ROCs. This can be explained as an effect of class imbalance, which is better leveraged by the PRC [21]. In the One‐vs‐Rest PRC curves (Figure 3d), we can easily identify the three underperforming curves (corresponding to CLCN1, HypoPP and SCN4A). Still, the micro‐ and macro‐averaged PRC curves suggest a good overall performance, with AUPRCs of 0.780 and 0.671 respectively. The performance metrics for each disease are shown in Table S1.

**FIGURE 4:**
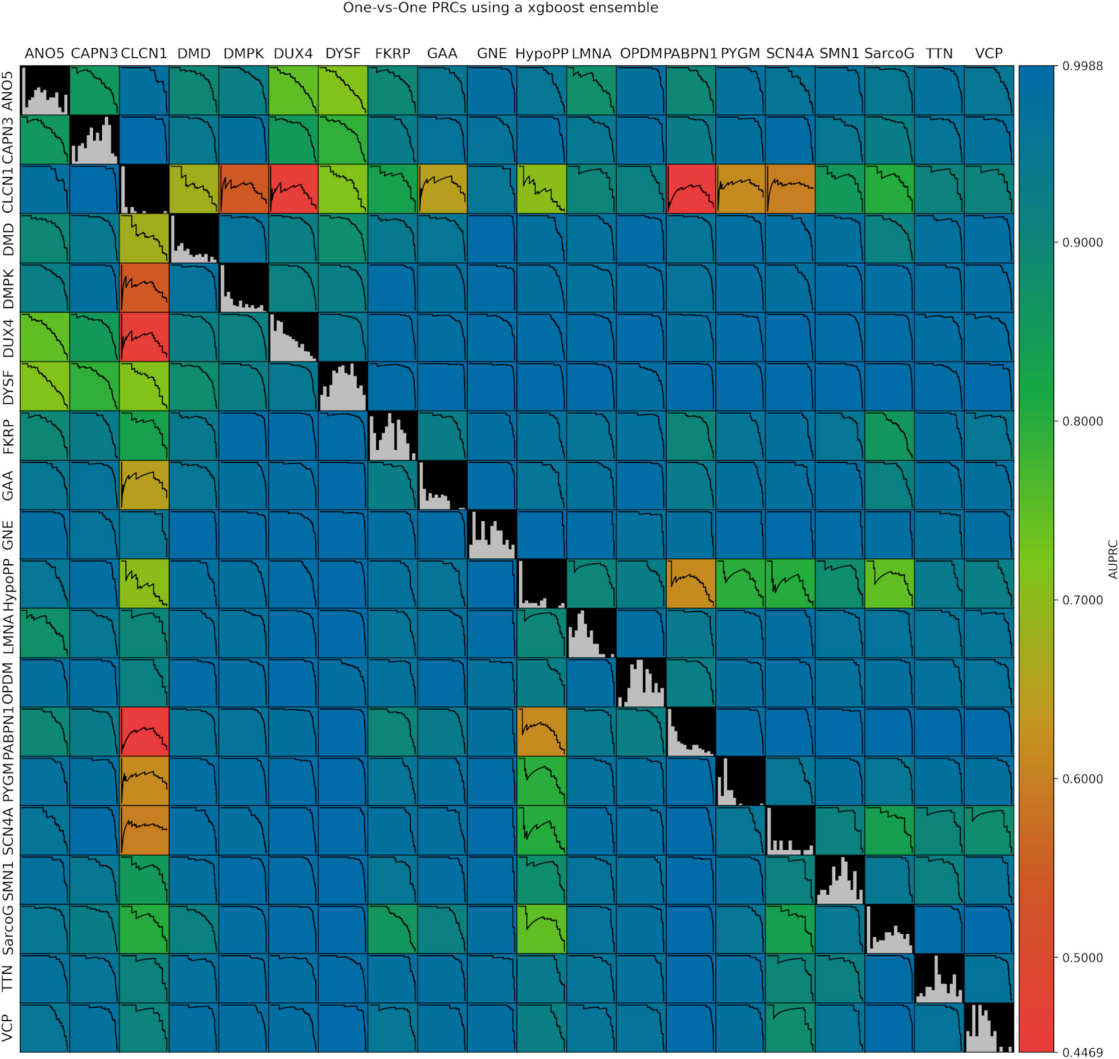
Average One‐vs‐One precision–recall curves. The areas under the precision–recall curves (AUPRC) are represented for each pair of diseases. Histograms of the mean fat score for each NMD are shown in the diagonal.

To further understand the performance of the model, we calculated the One‐vs‐One PRCs (Figure 4). Again, we can observe a lower performance when predicting CLCN1, HypoPP and SCN4A, especially when comparing them against diseases with a high positively skewed mean fat score distribution. We also observe how the model tends to confuse ANO5 and CAPN3 with DUX4 and DYSF. The latter two are the majority classes in the data and seem to bias the model, lowering the performance in predicting ANO5 and CAPN3. The rest of the diseases do not appear to be affected by this.

### 3.3 Classifier Comparison

Random forest achieved comparable results to XGBoost, obtaining a balanced accuracy of 64.3% ± 3.0%. KNN required significantly lower resources and time to train with minimal hyperparameter optimization and managed to achieve a balanced accuracy of 59.3% ± 2.3% (Figure S22).

### 3.4 Model Interpretation

The SHAP framework, grounded in the cooperative game theory concept of Shapley values, offers an intuitive and theoretically robust method for dissecting the decision‐making processes of various machine learning models [22]. It achieves this by attributing the contribution of each feature in the dataset to the model's prediction, unravelling the opaque inner workings of complex models.

Traditionally, the patterns of muscle involvement in different diseases have been described in an isolated way or within small disease groups. Although this method helps characterize the progression of a disease, patterns identified in this way lack the differential specificity to help diagnose NMD patients [14]. By using SHAP values, we can identify patterns of muscle involvement unique for each disease within the pool of NMDs used to train the diagnosis model. A more detailed explanation on SHAP and how it can be interpreted is available in the Data S2.

**FIGURE 5:**
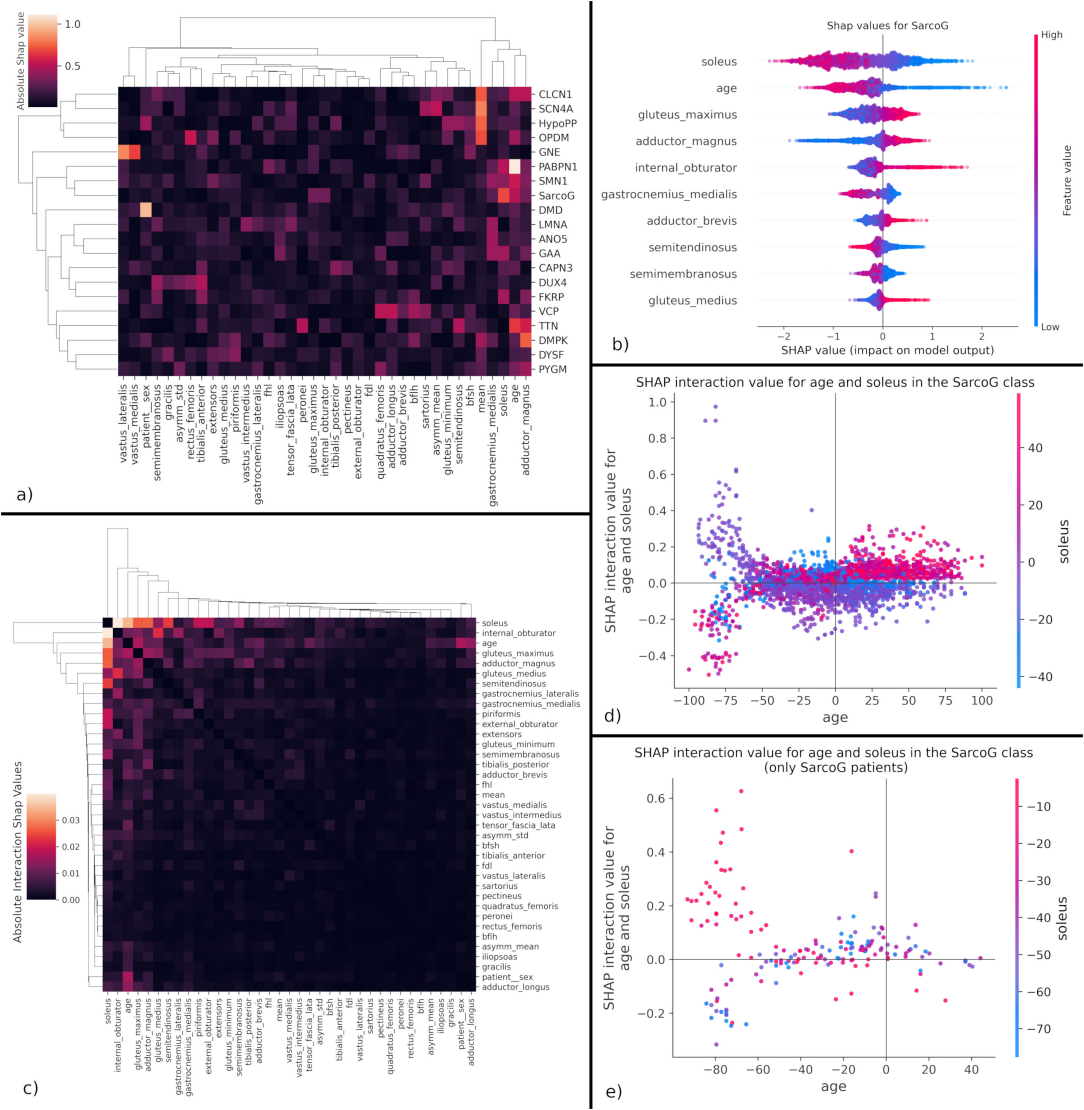
Model explainability using SHAP values. (a) Clustered mean absolute SHAP value. Features are shown in columns and diseases are shown in rows. The absolute SHAP value gives an overall indication of the importance of each feature. (b) SHAP values of the 10 most important features in predicting SarcoG, with each patient represented as a dot. Positive SHAP values indicate a positive impact on the prediction (increase in odds of predicting the target disease) and vice versa. Feature values are colour‐coded: ‘High’ is equivalent to the maximum feature value, and ‘low’ is equivalent to the minimum feature value. (c) Clustered mean absolute interaction values for SarcoG. The main effect values (diagonal) have been set to 0 to avoid obscuring the interaction values. (d) Interaction values between age and soleus for SarcoG, showing all patients. (e) Interaction values between age and soleus for SarcoG, only showing patients diagnosed with SarcoG (ground truth). Age is normalized to a −100–100 range.

In Figure 5a, we show a heatmap with the mean absolute SHAP value of each feature (columns), divided by each NMD (rows). We clustered the SHAP values to identify disease groups by feature importance. The model identifies two main groups: diseases characterized by the mean fat score (PYGM, DMPK, CLCN1, OPDM and SCN4A) and diseases characterized by the gastrocnemius medialis, soleus, age and adductor magnus (PABPN1, SMN1 and SarcoG). The vastus lateralis and medialis are especially relevant in diagnosing GNE. These muscles have been described to be completely spared in early and mid‐stage patients, showing involvement mostly in the final stage of the disease [23, 24].

We can further explore the importance of features for each disease by analysing the distribution of SHAP values within each disease and feature. Taking SarcoG as an example (Figure 5b), we can observe how having a high age is consistently associated with not having SarcoG, as indicated by the wide trail of negative shape values. In contrast, the long trail of positive SHAP values for the age feature suggests that having a low age is extremely important for some specific patients. SHAP values for all diseases are available in Figure S23.

Understanding the interactions between features can provide deeper insights into the characteristic patterns of each disease. SHAP allows us to calculate interaction values for each pair of features; however, analysing each pair of interactions becomes unpractical as the feature space increases. To approach this problem, we proposed clustering the mean absolute interaction values. For the SarcoG example (Figure 5c), we observe how the age, gluteus maximus and soleus have larger absolute interaction values, suggesting a synergy between these features. When analysing the interaction between soleus and age (Figure 5d,e), we observe that involvement of the soleus close to the average of the patient (values close to 0) at an early age increases the odds of diagnosing SarcoG. Although the scientific community has already identified a rapid‐progressing childhood variant and slow‐progressing adult‐onset variant for SarcoG [7, 25], we are, to the best of our knowledge, the first ones to link these variants to the soleus involvement as a discriminative feature for diagnosing SarcoG.

### 3.5 AI Versus Experts' Results

**FIGURE 6:**
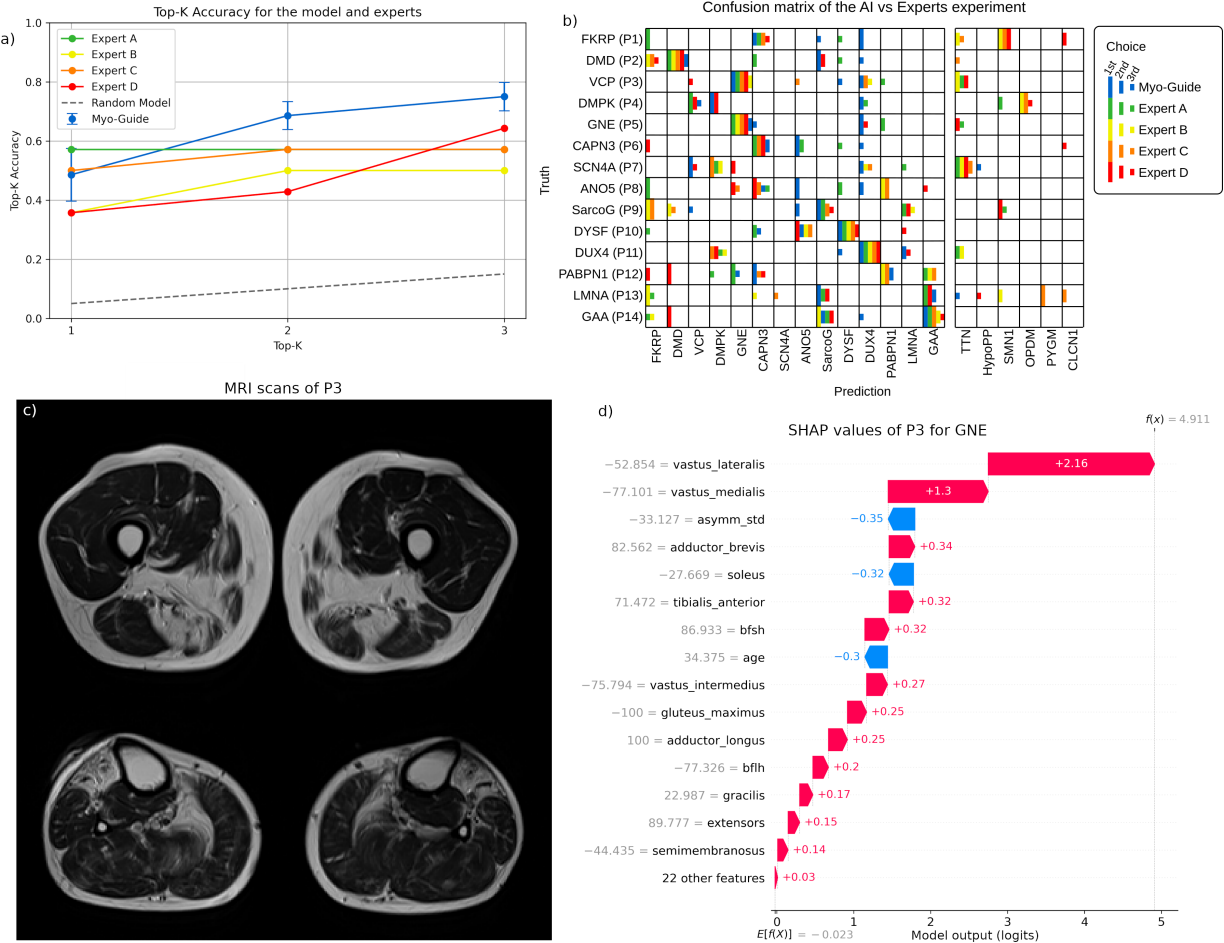
(a) Top‐K accuracy curves of the model (Myo‐Guide) and experts in the final AI versus experts' experiment. Error bars are available for the model, representing the standard deviation of the model ensemble. The Top‐K accuracy curve of a random classifier is also provided. (b) Confusion matrix of the AI versus experts' experiment, with each answer represented as a bar. Each expert (and model) is colour‐coded, and the ranking of each choice is represented by the length of the bar. The predicted diagnoses not included in the test set (TTN, HypoPP, SMN1, OPDM, PYGM and CLCN1) are separated from the rest for visual clarity. Bars in the diagonal represent correct predictions and vice versa. (c) mMRI scan of P3 showing thigh and lower leg. (d) Waterfall plot of the SHAP values for P3 when predicting GNE. The y‐axis represents the features (with values) sorted by decreasing importance (top to bottom). The x‐axis represents the raw output of the model (in logits). The plot shows the impact each feature had towards the model output. Note that all feature values are scaled to a range of −100 and 100 (including age and asymmetry).

In the comparative evaluation between the AI model and experienced clinicians in mMRI analysis, Myo‐Guide demonstrated the highest top‐3 accuracy (75.0% ± 4.8%), outperforming Expert D (64.3%) as shown in Figure 6a. Conversely, when considering only the primary prediction, the model ranked third (48.6% ± 8.9%), with Expert A achieving the highest accuracy (57.4%). A comprehensive breakdown of these results is provided in Figure 6b. Notably, both the model and the clinicians partially or entirely misclassified patients P1 (FKRP), P3 (VCP), P7 (SCN4A) and P13 (LMNA), suggesting that these cases exhibited atypical representations. In the case of patient P3, both the model and the experts consistently misdiagnosed the condition as GNE rather than VCP. An analysis of the SHAP values for patient P3's prediction revealed that the preservation of the anterior thigh compartment and the involvement of the posterior compartment were the primary contributing factors to this prediction.

### 3.6 Development of a Web Containing the Algorithm Described

The model has been deployed on the Myo‐Guide web platform, accessible at www.myoguide.org. The platform offers an interactive user interface to use the diagnosis model (Figure S24). No identifiable information is required, and no data are stored after submitting a request. The web deployment of the model allows its integration with clinical and genetic workflows, providing clinicians and experts with additional information helpful in the diagnosis process of patients.

## 4 Discussion

In this paper, we introduced a novel pipeline for processing and harmonizing multi‐study muscle fat replacement data obtained using MRI for diagnostic purposes. This advancement allowed us to gather the largest dataset published to date of muscle MRI data in NMD patients, enabling a new automated approach to the diagnosis of these disorders. With that objective in mind, we proposed a scalable methodology using AI and proved its performance in diagnosing patients encompassing 20 different diseases. We also adapted well‐established AI explainability techniques to explore distinctive patterns of muscle involvement, finding clues for diagnosis undescribed in previous research. Finally, we aimed to translate all these efforts into an immediate improvement in clinical practice by offering the model as a free web application.

We designed the complete pipeline to allow for different muscle fat scoring systems, with special care to quantitative measures such as the fat fraction. Radiology trends point towards an increase in the adoption of quantitative techniques, hence the importance of future‐proofing current technologies [26, 27]. Recent advancements in automatic muscle segmentation [28], together with the approaches presented here, are steps towards a streamlined approach to NMD diagnosis using MRI.

This processing pipeline also helps correct the potential effect of inter‐operator variability bias in qualitative scores. Scorers who tend to exaggerate fat involvement will (to some extent) score all muscles higher and vice versa. By using the mean of each patient to scale the scores, we convert the scores from an absolute measure of fat to a relative one, attenuating differences between scoring styles.

Still, there is a need for strategies enabling the reusability of retrospective data, allowing to automatically measure fat in T1w MRI scans and other imaging modalities. Different studies have proven the correlation between fat fraction and fat measurements obtained with other MRI techniques [29, 30], suggesting that such a system is possible.

In the same line as our work, Wei et al. [11] presented an AI model for differentiating necrotizing myopathy from dysferlinopathy using a mix of radiomic features and Mercuri fat scores, obtaining AUCs over 0.9. Nagawa et al. [12] presented several radiomics‐based models to classify patients diagnosed with dermatomyositis, amyopathic dermatomyositis and polymyositis, obtaining accuracies of approximately 61%. Monforte et al. [13] also presented a random forest model for differentiating between FSHD and non‐FSHD patients using Mercuri fat scores from upper and lower body muscles, obtaining an accuracy of 89%. Although current studies demonstrate the increasing role of AI in diagnosing NMD patients, these are still limited to a few diseases, and there is a lack of a standardized approach. We consider the pipeline presented in this paper as a breakthrough in this context, establishing best practices for muscle fat replacement data collection and standardization, design of automated diagnosis models and critical evaluation to avoid over‐optimistic performance metrics.

The applied AI explainability techniques provide an opportunity to reanalyse all the muscle fat data published during the last decades with a depth that was not possible before. Using our approach, muscle involvement can be characterized in the context of a large pool of diseases. Therefore, differential disease characteristics can be identified to aid in the diagnosis of these disorders. In the past, MRI was considered inaccurate in diagnosing some diseases, such as anoctaminopathy [31], due to the overlapping of muscle involvement patterns with other disorders [14]. Although our model has difficulties in diagnosing anoctaminopathy, we managed to predict more than half the anoctaminopathy patients correctly. Future studies should focus on applying the proposed explainability techniques to each one of the included diseases to expand our understanding of their clinical representations.

During the lifetime of this project, we observed the diagnosis model decay in performance as the number of diseases included increased. We reached a point where the addition of new samples did not necessarily translate into an improvement. Moreover, further increasing the number of samples for the majority classes might lower the performance of the model, as we demonstrated. The model could be improved by integrating more predictive features, such as upper‐body muscle fat data [32], muscle inflammation data obtained with fluid‐sensitive techniques such as the fat‐suppressed T2w or STIR MRI sequences [33] and clinical features.

The inclusion of disorders with minimal or no fatty replacement (CLCN1, HypoPP and SCN4A) accentuated the drop in performance when compared with our 2020 study, especially when considering balanced metrics. It could be argued that a patient diagnosed with one of these disorders is, from the model's perspective, no different than a patient with a pre‐symptomatic or early stage of a disorder where fatty replacement is common. Additionally, the lack of muscle involvement in patients who have muscle weakness in clinical examination has been defined as characteristic of some disorders, such as myasthenic syndromes or channelopathies [34, 35]. For these reasons, and to avoid cherry‐picking, we decided to keep these disorders in the dataset. One potential solution for a future version of the model could be to group all the diseases that have a normal or near‐normal MRI under the same category.

Other limitations of our approach are intrinsic to the features used for training the model. Muscle fat is not homogeneously distributed across the muscle volume, but there are different ‘textures’ of fat and patterns that can be useful for diagnosis [36]: popcorn fat (or fat pockets) in VCP [15], fat ring in Bethlem disease [37] and proximo‐distal distribution in sarcoglycanopathies [7], to enumerate a few. Mercuri‐based scales do not capture this information, as they approximate the whole muscle fat content as a single score. Dixon sequences could alleviate this by quantifying the complete muscle volume, although complex texture information could be captured using radiomics, an image analysis modality.

Also related with the latter limitation, this study challenges some accepted ideas on the patterns of muscle involvement, such as confusing SarcoG with FKRP more than with GAA. The presented model uses exclusively the overall amount of fatty replacement, age and sex; and it has no knowledge of fat distribution/texture, muscle hypertrophy/atrophy and other characteristics easily picked up by the expert's eye. The model has also no knowledge of the patient's family history, ethnic or geographic origin of the patient and other key clinical information. This leads to interesting results that challenge the descriptive papers published so far. By blinding the model to all other predictive features usually used in diagnostics, we can isolate the muscle involvement patterns from other predictive features observed both in and out of the MRI, allowing for a comprehensive understanding of these patterns.

Increasing the number of features would introduce another problem, as the complexity of collecting, curating and harmonizing the data would significantly increase [38]. Additionally, blocks of missing values would arise in the data as not all imaging modalities or body regions would be available for all patients. This has been solved here to an extent, but more specialized techniques might be necessary if the problem increases. This is an active topic of research, and multiple methods have been proposed over the last years to solve this problem [39, 40].

The detection of patients with a disorder outside of the presented pool of diseases (outlier detection) is not addressed in this study. Although classification and outlier detection are distinct tasks, the use of model confidence could provide insights into potential outliers. However, care must be taken to distinguish between patients with rare disease sub‐patterns and those with conditions not represented in the model. This remains an important area for future research, particularly in the context of neuromuscular diseases, where rare cases are common and clinically significant.

The final AI versus experts' experiment revealed not only the efficacy of the model in suggesting potential diagnoses. The concordance between the model and expert assessments in misclassified patients suggests that the AI system can identify differential patterns even in atypical cases. This information is key to ensure that the model predictions are kept grounded to clinically relevant features.

The proposed methods represent a leap forward in the way we use MRI for studying NMDs. By designing a flexible data standard and harmonization methods, we managed to conglomerate a large repository of rare disease data. This is an essential step in this new age of data‐driven approaches, which paves the way for a new wave of AI‐based strategies to study muscle MRI in NMDs. The presented online tool is especially useful for clinicians and geneticist, enhancing the decision‐making during the diagnosis pipeline with the outcomes of the AI‐based analysis. This tool can help with patients carrying a variant of unknown significance in a specific gene or patients who have multiple pathogenic variants in several genes. In those cases, having an MRI compatible with a specific pattern can help in the diagnosis process. Future work will expand on the methods proposed here, improving the diagnostic pipeline and deepening our understanding of these conditions.

## Ethics Statement

The authors affirm that this manuscript conforms to the journal guidelines for ethical publication.

## Conflicts of Interest

The authors declare no conflicts of interest.

## Supporting information


**Figure S1** Distribution of muscle fat scores before (a) and after (b) processing. Normalized stacked densities are shown for each different scale. The discrete values shown in Figure 1a are highlighted in the left figure.
**Figure S2:** Heatmap of the data for ANO5. Patient samples are represented in rows and features in columns. Rows are sorted by mean fat score, with late‐stage patients in the upper rows and early‐stage patients in the lower rows. Asymmetry is calculated as the difference between each left and right muscle, and the mean and standard deviation of all muscles are added as features to each patient. Muscle abbreviations: biceps femoris long head (bflh), biceps femoris short head (bfsh), flexor hallucis longus (fhl) and flexor digitorum longus (fdl). The extensor digitorum longus and extensor hallucis longus have been grouped and named ‘extensors’.
**Figure S3:** Heatmap of the data for CAPN3. Patient samples are represented in rows and features in columns. Rows are sorted by mean fat score, with late‐stage patients in the upper rows and early‐stage patients in the lower rows. Asymmetry is calculated as the difference between each left and right muscle, and the mean and standard deviation of all muscles are added as features to each patient. Muscle abbreviations: biceps femoris long head (bflh), biceps femoris short head (bfsh), flexor hallucis longus (fhl) and flexor digitorum longus (fdl). The extensor digitorum longus and extensor hallucis longus have been grouped and named ‘extensors’.
Figure S4: Heatmap of the data for CLCN1. Patient samples are represented in rows and features in columns. Rows are sorted by mean fat score, with late‐stage patients in the upper rows and early‐stage patients in the lower rows. Asymmetry is calculated as the difference between each left and right muscle, and the mean and standard deviation of all muscles are added as features to each patient. Muscle abbreviations: biceps femoris long head (bflh), biceps femoris short head (bfsh), flexor hallucis longus (fhl) and flexor digitorum longus (fdl). The extensor digitorum longus and extensor hallucis longus have been grouped and named ‘extensors’.
**Figure S5:** Heatmap of the data for DMD. Patient samples are represented in rows and features in columns. Rows are sorted by mean fat score, with late‐stage patients in the upper rows and early‐stage patients in the lower rows. Asymmetry is calculated as the difference between each left and right muscle, and the mean and standard deviation of all muscles are added as features to each patient. Muscle abbreviations: biceps femoris long head (bflh), biceps femoris short head (bfsh), flexor hallucis longus (fhl) and flexor digitorum longus (fdl). The extensor digitorum longus and extensor hallucis longus have been grouped and named ‘extensors’.
**Figure S6:** Heatmap of the data for DMPK. Patient samples are represented in rows and features in columns. Rows are sorted by mean fat score, with late‐stage patients in the upper rows and early‐stage patients in the lower rows. Asymmetry is calculated as the difference between each left and right muscle, and the mean and standard deviation of all muscles are added as features to each patient. Muscle abbreviations: biceps femoris long head (bflh), biceps femoris short head (bfsh), flexor hallucis longus (fhl) and flexor digitorum longus (fdl). The extensor digitorum longus and extensor hallucis longus have been grouped and named “extensors”.
**Figure S7:** Heatmap of the data for DUX4. Patient samples are represented in rows and features in columns. Rows are sorted by mean fat score, with late‐stage patients in the upper rows and early‐stage patients in the lower rows. Asymmetry is calculated as the difference between each left and right muscle, and the mean and standard deviation of all muscles are added as features to each patient. Muscle abbreviations: biceps femoris long head (bflh), biceps femoris short head (bfsh), flexor hallucis longus (fhl) and flexor digitorum longus (fdl). The extensor digitorum longus and extensor hallucis longus have been grouped and named ‘extensors’.
**Figure S8:** Heatmap of the data for DYSF. Patient samples are represented in rows and features in columns. Rows are sorted by mean fat score, with late‐stage patients in the upper rows and early‐stage patients in the lower rows. Asymmetry is calculated as the difference between each left and right muscle, and the mean and standard deviation of all muscles are added as features to each patient. Muscle abbreviations: biceps femoris long head (bflh), biceps femoris short head (bfsh), flexor hallucis longus (fhl) and flexor digitorum longus (fdl). The extensor digitorum longus and extensor hallucis longus have been grouped and named ‘extensors’.
**Figure S9:** Heatmap of the data for FKRP. Patient samples are represented in rows and features in columns. Rows are sorted by mean fat score, with late‐stage patients in the upper rows and early‐stage patients in the lower rows. Asymmetry is calculated as the difference between each left and right muscle, and the mean and standard deviation of all muscles are added as features to each patient. Muscle abbreviations: biceps femoris long head (bflh), biceps femoris short head (bfsh), flexor hallucis longus (fhl) and flexor digitorum longus (fdl). The extensor digitorum longus and extensor hallucis longus have been grouped and named ‘extensors’.
**Figure S10:** Heatmap of the data for GAA. Patient samples are represented in rows and features in columns. Rows are sorted by mean fat score, with late‐stage patients in the upper rows and early‐stage patients in the lower rows. Asymmetry is calculated as the difference between each left and right muscle, and the mean and standard deviation of all muscles are added as features to each patient. Muscle abbreviations: biceps femoris long head (bflh), biceps femoris short head (bfsh), flexor hallucis longus (fhl) and flexor digitorum longus (fdl). The extensor digitorum longus and extensor hallucis longus have been grouped and named ‘extensors’.
**Figure S11:** Heatmap of the data for GNE. Patient samples are represented in rows and features in columns. Rows are sorted by mean fat score, with late‐stage patients in the upper rows and early‐stage patients in the lower rows. Asymmetry is calculated as the difference between each left and right muscle, and the mean and standard deviation of all muscles are added as features to each patient. Muscle abbreviations: biceps femoris long head (bflh), biceps femoris short head (bfsh), flexor hallucis longus (fhl) and flexor digitorum longus (fdl). The extensor digitorum longus and extensor hallucis longus have been grouped and named ‘extensors’.
**Figure S12:** Heatmap of the data for HypoPP. Patient samples are represented in rows and features in columns. Rows are sorted by mean fat score, with late‐stage patients in the upper rows and early‐stage patients in the lower rows. Asymmetry is calculated as the difference between each left and right muscle, and the mean and standard deviation of all muscles are added as features to each patient. Muscle abbreviations: biceps femoris long head (bflh), biceps femoris short head (bfsh), flexor hallucis longus (fhl) and flexor digitorum longus (fdl). The extensor digitorum longus and extensor hallucis longus have been grouped and named ‘extensors’.
**Figure S13:** Heatmap of the data for LMNA. Patient samples are represented in rows and features in columns. Rows are sorted by mean fat score, with late‐stage patients in the upper rows and early‐stage patients in the lower rows. Asymmetry is calculated as the difference between each left and right muscle, and the mean and standard deviation of all muscles are added as features to each patient. Muscle abbreviations: biceps femoris long head (bflh), biceps femoris short head (bfsh), flexor hallucis longus (fhl) and flexor digitorum longus (fdl). The extensor digitorum longus and extensor hallucis longus have been grouped and named ‘extensors’.
**Figure S14:** Heatmap of the data for OPDM. Patient samples are represented in rows and features in columns. Rows are sorted by mean fat score, with late‐stage patients in the upper rows and early‐stage patients in the lower rows. Asymmetry is calculated as the difference between each left and right muscle, and the mean and standard deviation of all muscles are added as features to each patient. Muscle abbreviations: biceps femoris long head (bflh), biceps femoris short head (bfsh), flexor hallucis longus (fhl) and flexor digitorum longus (fdl). The extensor digitorum longus and extensor hallucis longus have been grouped and named ‘extensors’.
**Figure S15:** Heatmap of the data for PABPN1. Patient samples are represented in rows and features in columns. Rows are sorted by mean fat score, with late‐stage patients in the upper rows and early‐stage patients in the lower rows. Asymmetry is calculated as the difference between each left and right muscle, and the mean and standard deviation of all muscles are added as features to each patient. Muscle abbreviations: biceps femoris long head (bflh), biceps femoris short head (bfsh), flexor hallucis longus (fhl) and flexor digitorum longus (fdl). The extensor digitorum longus and extensor hallucis longus have been grouped and named ‘extensors’.
**Figure S16:** Heatmap of the data for PYGM. Patient samples are represented in rows and features in columns. Rows are sorted by mean fat score, with late‐stage patients in the upper rows and early‐stage patients in the lower rows. Asymmetry is calculated as the difference between each left and right muscle, and the mean and standard deviation of all muscles are added as features to each patient. Muscle abbreviations: biceps femoris long head (bflh), biceps femoris short head (bfsh), flexor hallucis longus (fhl) and flexor digitorum longus (fdl). The extensor digitorum longus and extensor hallucis longus have been grouped and named ‘extensors’.
**Figure S17:** Heatmap of the data for SCN4A. Patient samples are represented in rows and features in columns. Rows are sorted by mean fat score, with late‐stage patients in the upper rows and early‐stage patients in the lower rows. Asymmetry is calculated as the difference between each left and right muscle, and the mean and standard deviation of all muscles are added as features to each patient. Muscle abbreviations: biceps femoris long head (bflh), biceps femoris short head (bfsh), flexor hallucis longus (fhl) and flexor digitorum longus (fdl). The extensor digitorum longus and extensor hallucis longus have been grouped and named ‘extensors’.
**Figure S18:** Heatmap of the data for SMN1. Patient samples are represented in rows and features in columns. Rows are sorted by mean fat score, with late‐stage patients in the upper rows and early‐stage patients in the lower rows. Asymmetry is calculated as the difference between each left and right muscle, and the mean and standard deviation of all muscles are added as features to each patient. Muscle abbreviations: biceps femoris long head (bflh), biceps femoris short head (bfsh), flexor hallucis longus (fhl) and flexor digitorum longus (fdl). The extensor digitorum longus and extensor hallucis longus have been grouped and named ‘extensors’.
**Figure S19:** Heatmap of the data for TTN. Patient samples are represented in rows and features in columns. Rows are sorted by mean fat score, with late‐stage patients in the upper rows and early‐stage patients in the lower rows. Asymmetry is calculated as the difference between each left and right muscle, and the mean and standard deviation of all muscles are added as features to each patient. Muscle abbreviations: biceps femoris long head (bflh), biceps femoris short head (bfsh), flexor hallucis longus (fhl) and flexor digitorum longus (fdl). The extensor digitorum longus and extensor hallucis longus have been grouped and named ‘extensors’.
**Figure S20:** Heatmap of the data for VCP. Patient samples are represented in rows and features in columns. Rows are sorted by mean fat score, with late‐stage patients in the upper rows and early‐stage patients in the lower rows. Asymmetry is calculated as the difference between each left and right muscle, and the mean and standard deviation of all muscles are added as features to each patient. Muscle abbreviations: biceps femoris long head (bflh), biceps femoris short head (bfsh), flexor hallucis longus (fhl) and flexor digitorum longus (fdl). The extensor digitorum longus and extensor hallucis longus have been grouped and named ‘extensors’.
**Figure S21:** Distribution of the average muscle fat score for each disease with median values shown in red.
**Table S1:** Performance of the model ensemble for each disease.
**Figure S22:** Weighted top‐N accuracy as a function of N for each classifier. When *N* = 1, the weighted top‐N accuracy is equivalent to the balanced accuracy. When *N* = 20, the weighted top‐N accuracy achieves perfection and is meaningless, as all classes are considered in the calculation of the metric.
**Figure S23:** SHAP values of the 10 most important features in predicting each disease. Each subplot corresponds to a different disease. Positive SHAP values indicate a positive impact on the prediction (increase in odds of predicting the target disease) and vice versa. Feature values are colour‐coded: ‘High’ is equivalent to the maximum feature value, and ‘low’ is equivalent to the minimum feature value.
**Figure S24:** Screenshot of the interactive user interface of the web deployment. The platform offers an interactive representation of the leg muscles, allowing the user to score each patient visually.

**Data S1** Supporting Information.

## Data Availability

The corresponding author can facilitate a list of the publications with data included in this work. Unpublished data are not eligible for sharing due to a lack of consent from patients. Most of the dysferlinopathy data belong to the Jain COS project, available at www.jain‐foundation.org. The code used for this project is published under the myoguide‐diagnose python package, available at www.github.com/MYO‐Guide/myoguide‐diagnose .
