# Supplementary material for: Myo‐Guide: A Machine Learning‐Based Web Application for Neuromuscular Disease Diagnosis With MRI
Source: J Cachexia Sarcopenia Muscle. 2025 Apr 24;16(3):e13815. doi: 10.1002/jcsm.13815 (PMC12022233; doi:10.1002/jcsm.13815)
Supplement: Supplementary file 1 — Figure S1 Distribution of muscle fat scores before (a) and after (b) processing. Normalized stacked densities are shown for each different scale. The discrete values shown in Figure 1a are highlighted in the left figure. Figure S2: Heatmap of the data for ANO5. Patient samples are represented in rows and features in columns. Rows are sorted by mean fat score, with late‐stage patients in the upper rows and early‐stage patients in the lower rows. Asymmetry is calculated as the difference between each left and right muscle, and the mean and standard deviation of all muscles are added as features to each patient. Muscle abbreviations: biceps femoris long head (bflh), biceps femoris short head (bfsh), flexor hallucis longus (fhl) and flexor digitorum longus (fdl). The extensor digitorum longus and extensor hallucis longus have been grouped and named ‘extensors’. Figure S3: Heatmap of the data for CAPN3. Patient samples are represented in rows and features in columns. Rows are sorted by mean fat score, with late‐stage patients in the upper rows and early‐stage patients in the lower rows. Asymmetry is calculated as the difference between each left and right muscle, and the mean and standard deviation of all muscles are added as features to each patient. Muscle abbreviations: biceps femoris long head (bflh), biceps femoris short head (bfsh), flexor hallucis longus (fhl) and flexor digitorum longus (fdl). The extensor digitorum longus and extensor hallucis longus have been grouped and named ‘extensors’. Figure S4: Heatmap of the data for CLCN1. Patient samples are represented in rows and features in columns. Rows are sorted by mean fat score, with late‐stage patients in the upper rows and early‐stage patients in the lower rows. Asymmetry is calculated as the difference between each left and right muscle, and the mean and standard deviation of all muscles are added as features to each patient. Muscle abbreviations: biceps femoris long head (bflh), biceps femoris short head (b [file JCSM-16-e13815-s001.docx]

# Supplementary File 1

**Supplementary References**

S1. Sarkozy A, Deschauer M, Carlier RY, et al. Muscle MRI findings in limb girdle muscular dystrophy type 2L. *Neuromuscular Disorders*. 2012;22:S122-S129

S2. Tasca G, Iannaccone E, Monforte M, et al. Muscle MRI in Becker muscular dystrophy. *Neuromuscular Disorders*. 2012;22:S100-S106

S3. Tasca G, Monforte M, Díaz-Manera J, et al. MRI in sarcoglycanopathies: a large international cohort study. *J Neurol Neurosurg Psychiatry*. 2018;89(1):72-77

S4. Alonso-Jimenez A, Kroon RHMJM, Alejaldre-Monforte A, et al. Muscle MRI in a large cohort of patients with oculopharyngeal muscular dystrophy. *Journal of Neurology, Neurosurgery and Psychiatry*. 2019;90(5):576

S5. Murphy AP, Morrow J, Dahlqvist JR, et al. Natural history of limb girdle muscular dystrophy R9 over 6 years: searching for trial endpoints. *Ann Clin Transl Neurol*. 2019;6(6):1033-1045

S6. Brogna C, Cristiano L, Verdolotti T, et al. MRI patterns of muscle involvement in type 2 and 3 spinal muscular atrophy patients. *J Neurol*. 2020;267(4):898-912

S7. Díaz-Manera J, Alejaldre A, González L, et al. Muscle imaging in muscle dystrophies produced by mutations in the EMD and LMNA genes. *Neuromuscular Disorders*. 2016;26(1):33-40

S8. Willis TA, Hollingsworth KG, Coombs A, et al. Quantitative Muscle MRI as an Assessment Tool for Monitoring Disease Progression in LGMD2I: A Multicentre Longitudinal Study. *PLoS One*. 2013;8(8):e70993-

S9. Eura N, Noguchi S, Ogasawara M, et al. Characteristics of the muscle involvement along the disease progression in a large cohort of oculopharyngodistal myopathy compared to oculopharyngeal muscular dystrophy. *J Neurol*. 2023;270(12):5988-5998

S10. Garibaldi M, Nicoletti T, Bucci E, et al. Muscle magnetic resonance imaging in myotonic dystrophy type 1 (DM1): Refining muscle involvement and implications for clinical trials. *Eur J Neurol*. 2022;29(3):843-854

S11. Barp A, Laforet P, Bello L, et al. European muscle MRI study in limb girdle muscular dystrophy type R1/2A (LGMDR1/LGMD2A). *J Neurol*. 2020;267(1):45-56

S12. Tasca G, Ricci E, Monforte M, et al. Muscle imaging findings in GNE myopathy. *J Neurol*. 2012;259(7):1358-1365

S13. Kroon RHMJM, Kalf JG, de Swart BJM, et al. Muscle MRI in Patients With Oculopharyngeal Muscular Dystrophy. *Neurology*. 2024;102(1):e207833

S14. Gómez-Andrés D, Dabaj I, Mompoint D, et al. Pediatric laminopathies: Whole-body magnetic resonance imaging fingerprint and comparison with Sepn1 myopathy. *Muscle Nerve*. 2016;54(2):192-202

S15. Xie Z, Xie Z, Yu M, et al. Value of muscle magnetic resonance imaging in the differential diagnosis of muscular dystrophies related to the dystrophin-glycoprotein complex. *Orphanet J Rare Dis*. 2019;14(1):250

S16. Mul K, Vincenten SCC, Voermans NC, et al. Adding quantitative muscle MRI to the FSHD clinical trial toolbox. *Neurology*. 2017;89(20):2057-2065

S17. Leung DG, Carrino JA, Wagner KR, Jacobs MA. Whole-body magnetic resonance imaging evaluation of facioscapulohumeral muscular dystrophy. *Muscle Nerve*. 2015;52(4):512-520

S18. Løkken N, Revsbech KL, Jacobsen LN, et al. Muscle MRI in McArdle Disease. *Neurology*. 2022;99(15):e1664-e1675

S19. Shi Y, Cao C, Zeng Y, et al. CGG repeat expansion in LOC642361/NUTM2B-AS1 typically presents as oculopharyngodistal myopathy. *Journal of Genetics and Genomics*. 2024;51(2):184-196

S20. Barp A, Bello L, Caumo L, et al. Muscle MRI and functional outcome measures in Becker muscular dystrophy. *Sci Rep*. 2017;7(1):16060

S21. Fatehi F, Advani S, Okhovat AA, Ziaadini B, Shamshiri H, Nafissi S. Thigh and Leg Muscle MRI Findings in GNE Myopathy. *J Neuromuscul Dis*. 2021;8:735-742

S22. Aivazoglou LU, Guimarães JB, Costa MAF, et al. Whole-Body MRI in Limb Girdle Muscular Dystrophy Type R1/2A: Correlation With Clinical Scores. *Muscle Nerve*. 2022;66(4):471-478

S23. Forsting J, Rohm M, Froeling M, et al. Quantitative muscle MRI captures early muscle degeneration in calpainopathy. *Sci Rep*. 2022;12(1):19676

S24. Fischmann A, Gloor M, Fasler S, et al. Muscular involvement assessed by MRI correlates to motor function measurement values in oculopharyngeal muscular dystrophy. J Neurol. 2011;258(7):1333-1340

S25. Dong X, Lin L, Zhang R, et al. TOBMI: trans-omics block missing data imputation using a k-nearest neighbor weighted approach. Bioinformatics. 2019;35(8):1278-1283

S26. Thomas T, Rajabi E. Addressing Missing Data in a Healthcare Dataset Using an Improved kNN Algorithm. In: Paszynski M, Kranzlmüller D, Krzhizhanovskaya V V, Dongarra JJ, Sloot PMA, eds. Computational Science – ICCS 2021. Springer International Publishing; 2021:223-230.

S27. Chawla N V, Bowyer KW, Hall LO, Kegelmeyer WP. SMOTE: Synthetic Minority Over-sampling Technique. Journal of Artificial Intelligence Research. 2002;16:321-357

S28. Wu J, Chen XY, Zhang H, Xiong LD, Lei H, Deng SH. Hyperparameter Optimization for Machine Learning Models Based on Bayesian Optimization. Journal of Electronic Science and Technology. 2019;17(1):26-40

S29. Watanabe S. Tree-Structured Parzen Estimator: Understanding Its Algorithm Components and Their Roles for Better Empirical Performance. Published online 2023.

S30. Akiba T, Sano S, Yanase T, Ohta T, Koyama M. Optuna: A Next-generation Hyperparameter Optimization Framework. In: Proceedings of the 25th ACM SIGKDD International Conference on Knowledge Discovery & Data Mining. KDD ’19. Association for Computing Machinery; 2019:2623-2631

**Supplementary Figures**

#
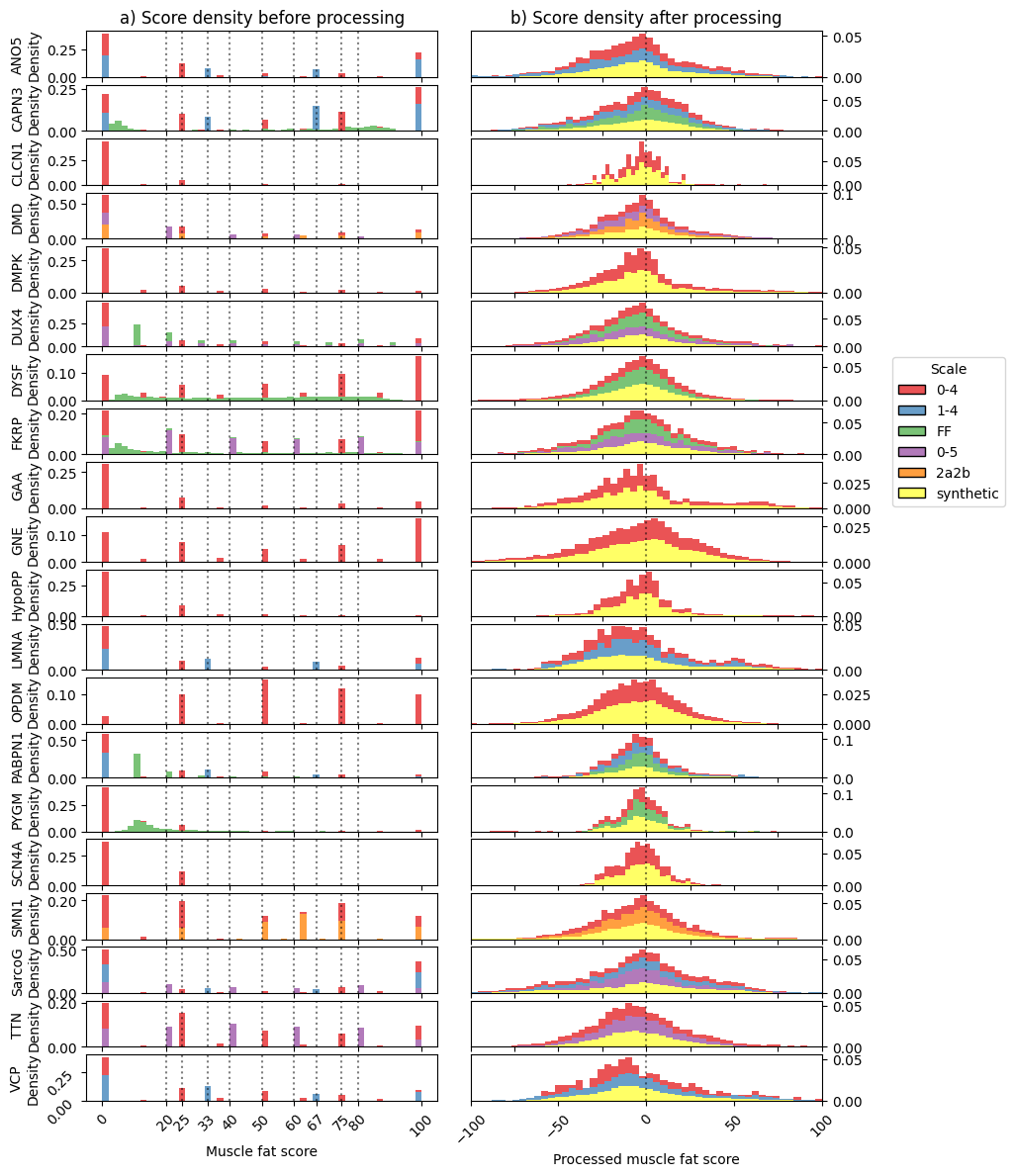


# Fig. S1: Distribution of muscle fat scores before (a) and after (b) processing. Normalized stacked densities are shown for each different scale. The discrete values shown in Fig. 1a are highlighted in the left figure.

#
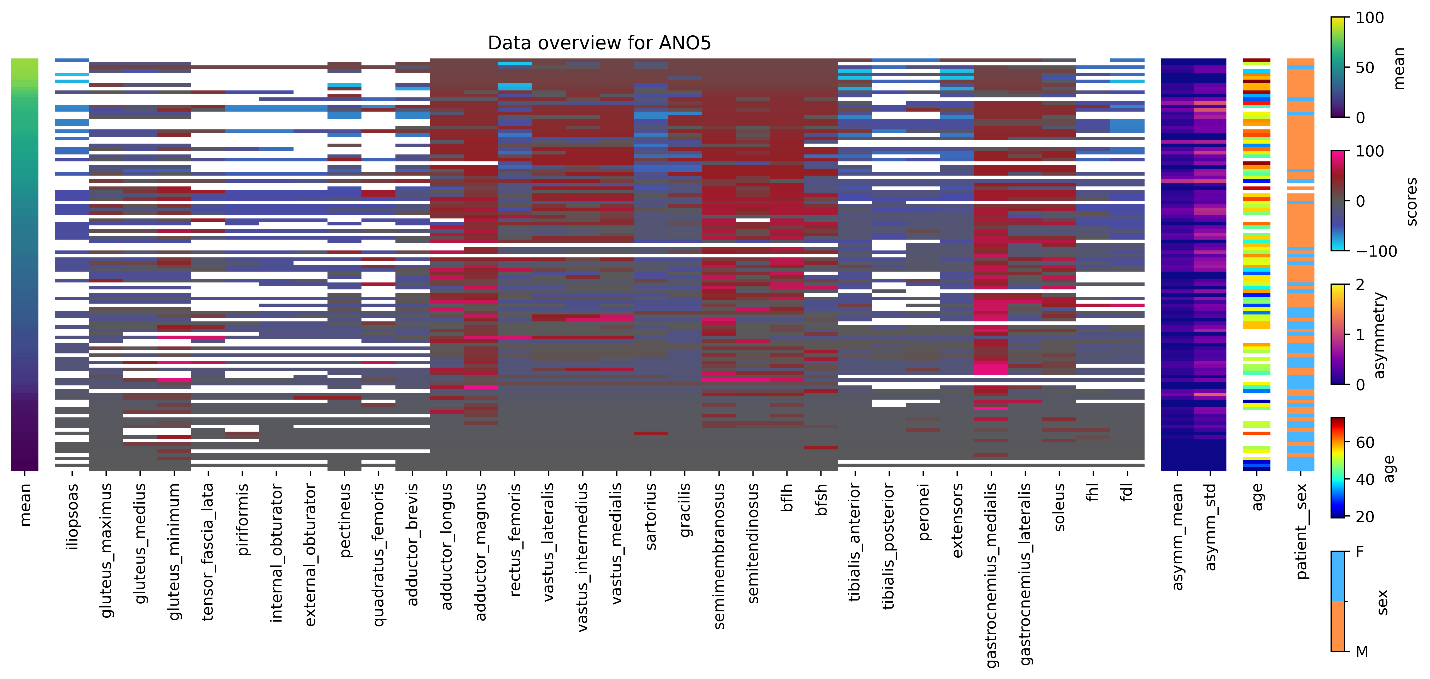
Fig. S2: Heatmap of the data for ANO5. Patient samples are represented in rows and features in columns. Rows are sorted by mean fat score, with late-stage patients in the upper rows and early-stage patients in the lower rows. Asymmetry is calculated as the difference between each left and right muscle, and the mean and standard deviation of all muscles are added as features to each patient. Muscle abbreviations: biceps femoris long head (bflh), biceps femoris short head (bfsh), flexor hallucis longus (fhl), flexor digitorum longus (fdl). The extensor digitorum longus and extensor hallucis longus have been grouped and named “extensors”.

#
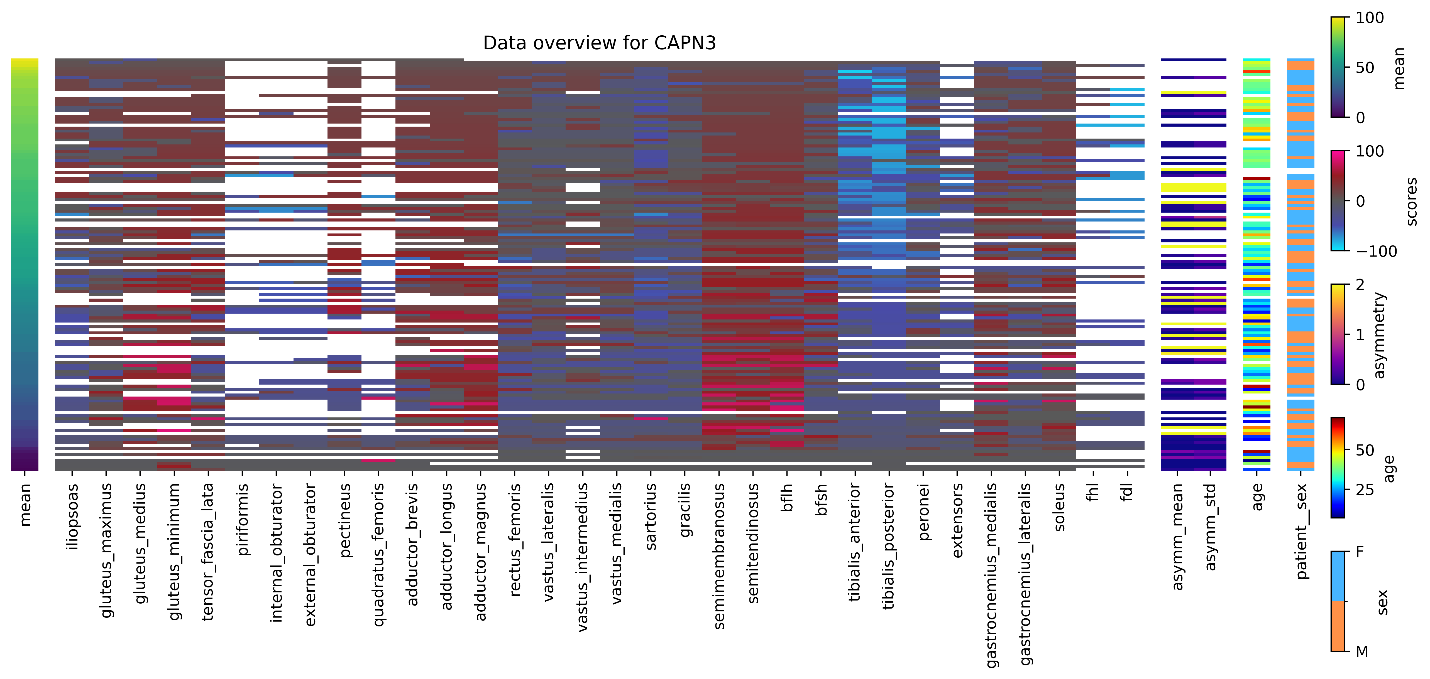
Fig. S3: Heatmap of the data for CAPN3. Patient samples are represented in rows and features in columns. Rows are sorted by mean fat score, with late-stage patients in the upper rows and early-stage patients in the lower rows. Asymmetry is calculated as the difference between each left and right muscle, and the mean and standard deviation of all muscles are added as features to each patient. Muscle abbreviations: biceps femoris long head (bflh), biceps femoris short head (bfsh), flexor hallucis longus (fhl), flexor digitorum longus (fdl). The extensor digitorum longus and extensor hallucis longus have been grouped and named “extensors”.

#
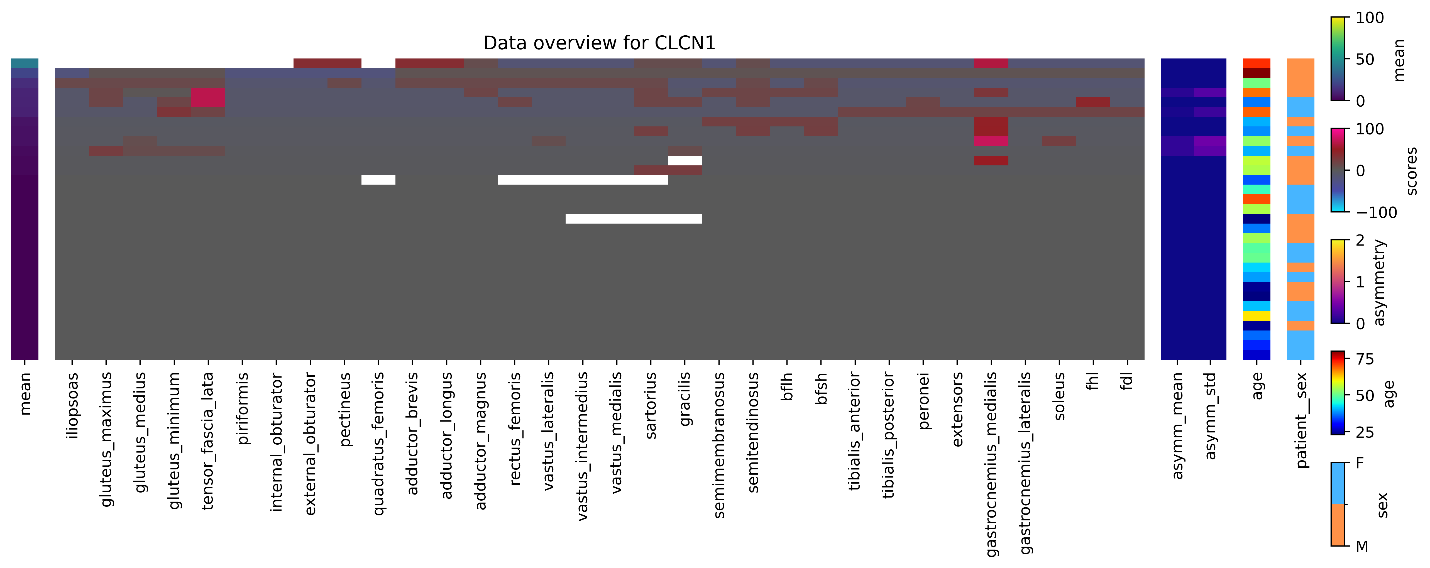
Fig. S4: Heatmap of the data for CLCN1. Patient samples are represented in rows and features in columns. Rows are sorted by mean fat score, with late-stage patients in the upper rows and early-stage patients in the lower rows. Asymmetry is calculated as the difference between each left and right muscle, and the mean and standard deviation of all muscles are added as features to each patient. Muscle abbreviations: biceps femoris long head (bflh), biceps femoris short head (bfsh), flexor hallucis longus (fhl), flexor digitorum longus (fdl). The extensor digitorum longus and extensor hallucis longus have been grouped and named “extensors”.

#
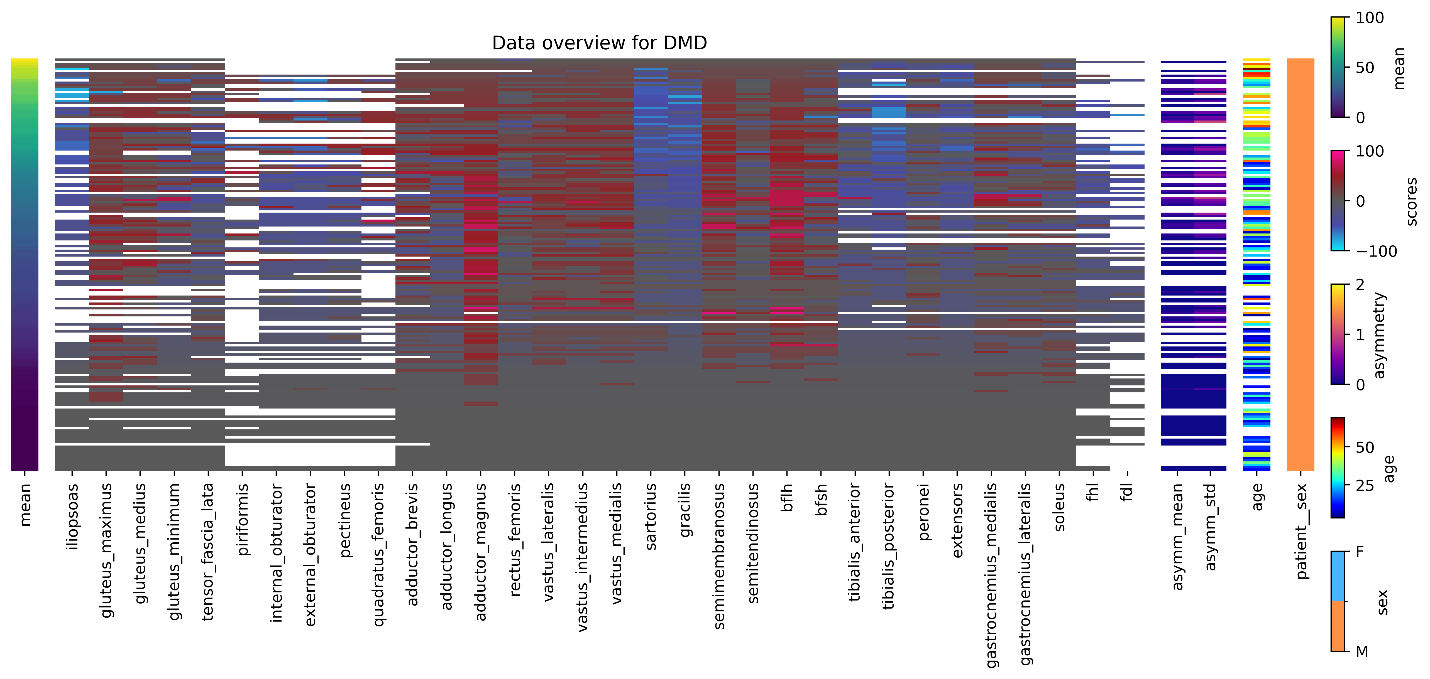
Fig. S5: Heatmap of the data for DMD. Patient samples are represented in rows and features in columns. Rows are sorted by mean fat score, with late-stage patients in the upper rows and early-stage patients in the lower rows. Asymmetry is calculated as the difference between each left and right muscle, and the mean and standard deviation of all muscles are added as features to each patient. Muscle abbreviations: biceps femoris long head (bflh), biceps femoris short head (bfsh), flexor hallucis longus (fhl), flexor digitorum longus (fdl). The extensor digitorum longus and extensor hallucis longus have been grouped and named “extensors”.

#
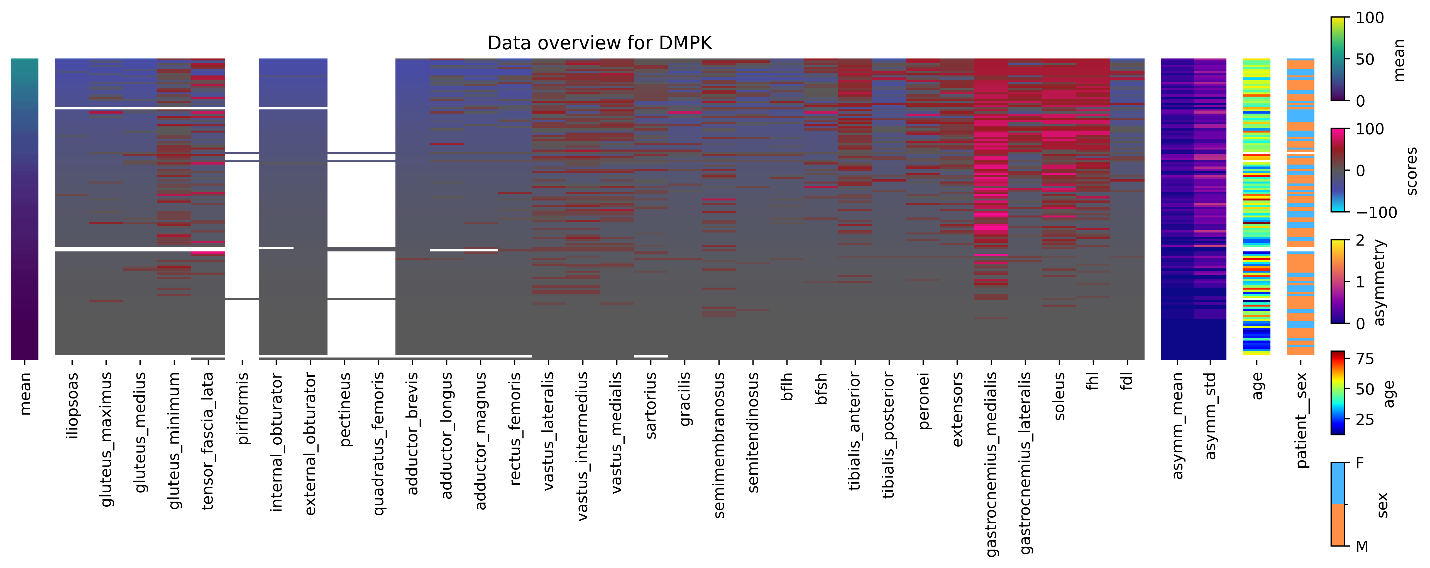
Fig. S6: Heatmap of the data for DMPK. Patient samples are represented in rows and features in columns. Rows are sorted by mean fat score, with late-stage patients in the upper rows and early-stage patients in the lower rows. Asymmetry is calculated as the difference between each left and right muscle, and the mean and standard deviation of all muscles are added as features to each patient. Muscle abbreviations: biceps femoris long head (bflh), biceps femoris short head (bfsh), flexor hallucis longus (fhl), flexor digitorum longus (fdl). The extensor digitorum longus and extensor hallucis longus have been grouped and named “extensors”.

#
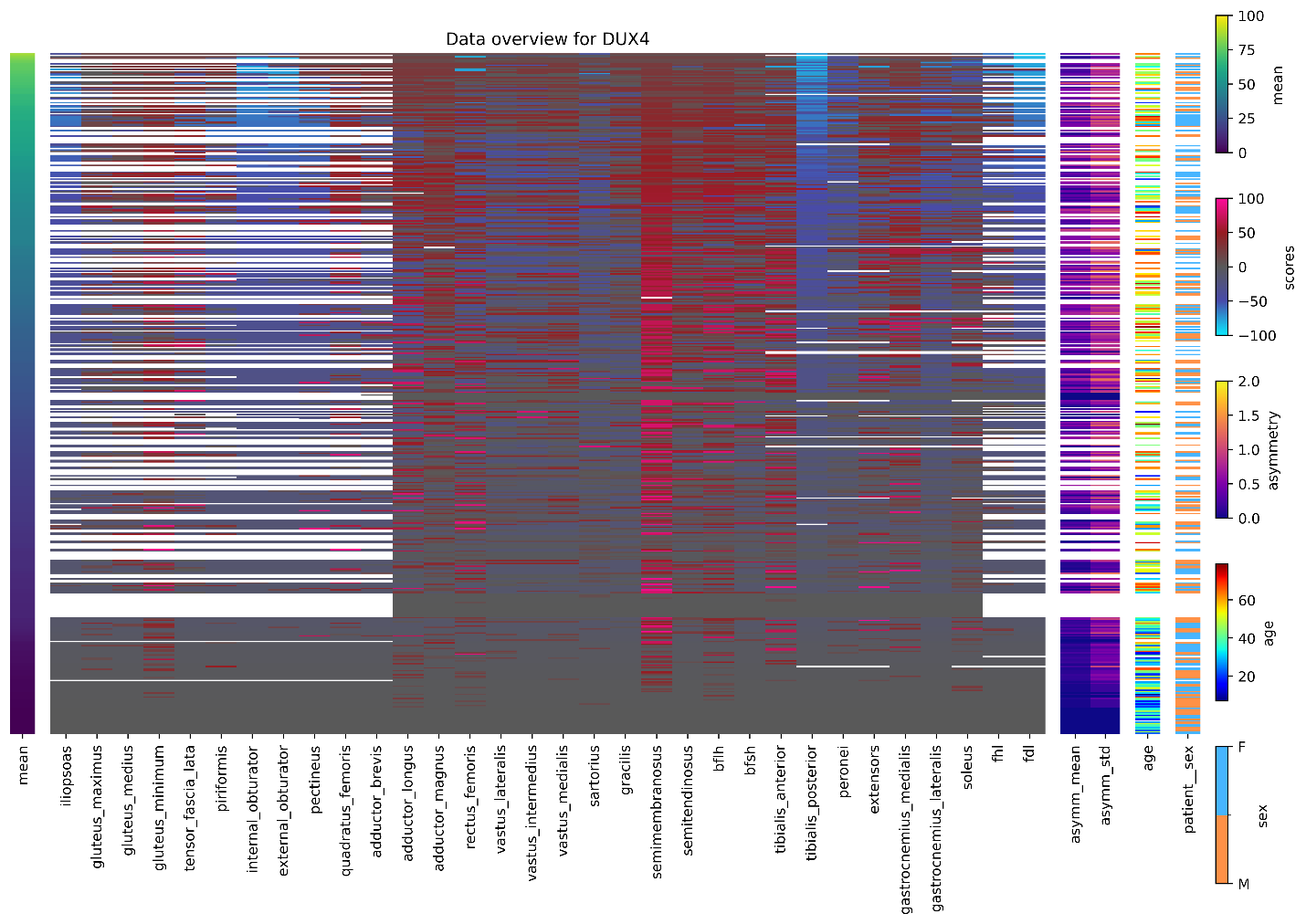
Fig. S7: Heatmap of the data for DUX4. Patient samples are represented in rows and features in columns. Rows are sorted by mean fat score, with late-stage patients in the upper rows and early-stage patients in the lower rows. Asymmetry is calculated as the difference between each left and right muscle, and the mean and standard deviation of all muscles are added as features to each patient. Muscle abbreviations: biceps femoris long head (bflh), biceps femoris short head (bfsh), flexor hallucis longus (fhl), flexor digitorum longus (fdl). The extensor digitorum longus and extensor hallucis longus have been grouped and named “extensors”.

#
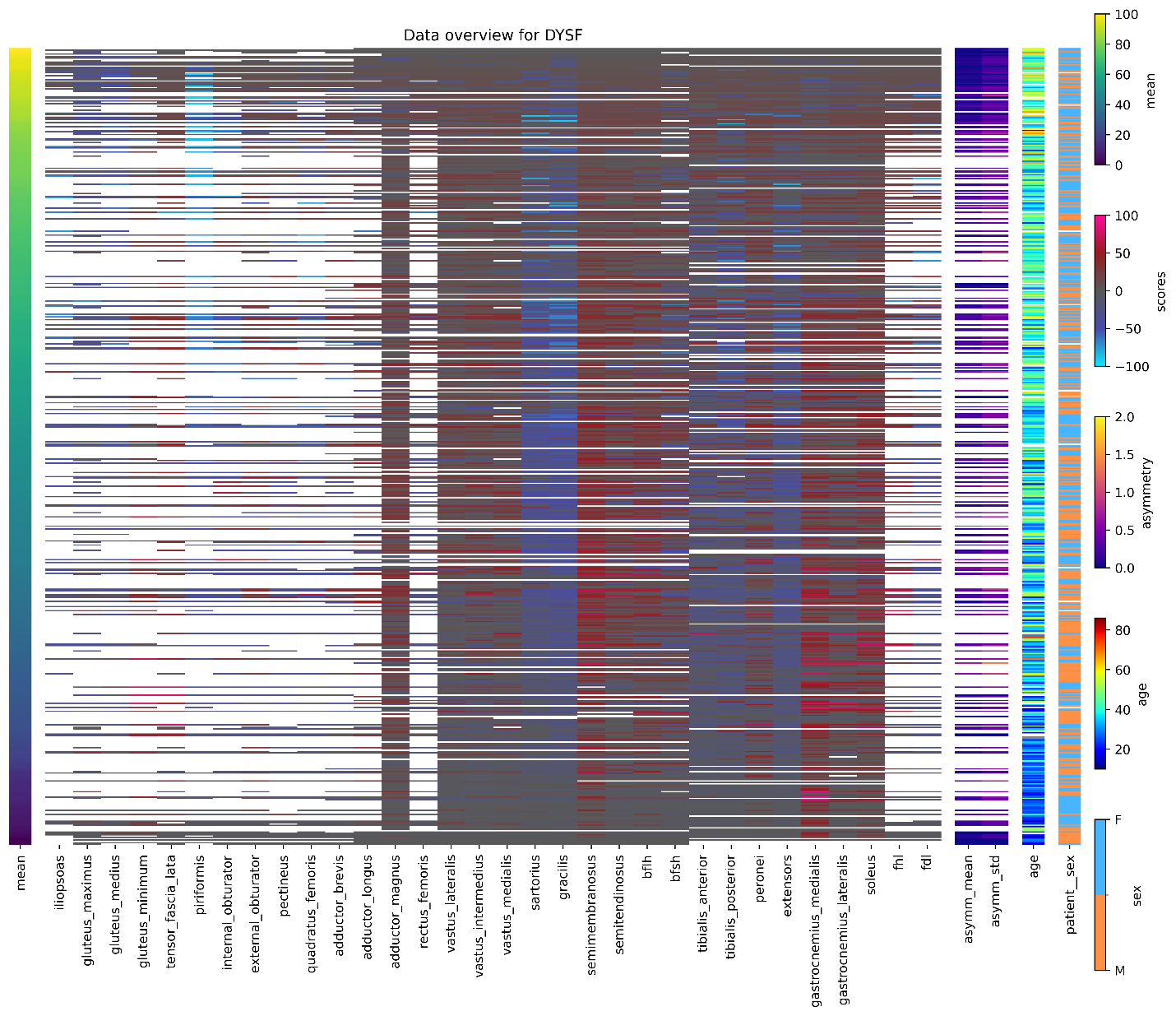
Fig. S8: Heatmap of the data for DYSF. Patient samples are represented in rows and features in columns. Rows are sorted by mean fat score, with late-stage patients in the upper rows and early-stage patients in the lower rows. Asymmetry is calculated as the difference between each left and right muscle, and the mean and standard deviation of all muscles are added as features to each patient. Muscle abbreviations: biceps femoris long head (bflh), biceps femoris short head (bfsh), flexor hallucis longus (fhl), flexor digitorum longus (fdl). The extensor digitorum longus and extensor hallucis longus have been grouped and named “extensors”.

#
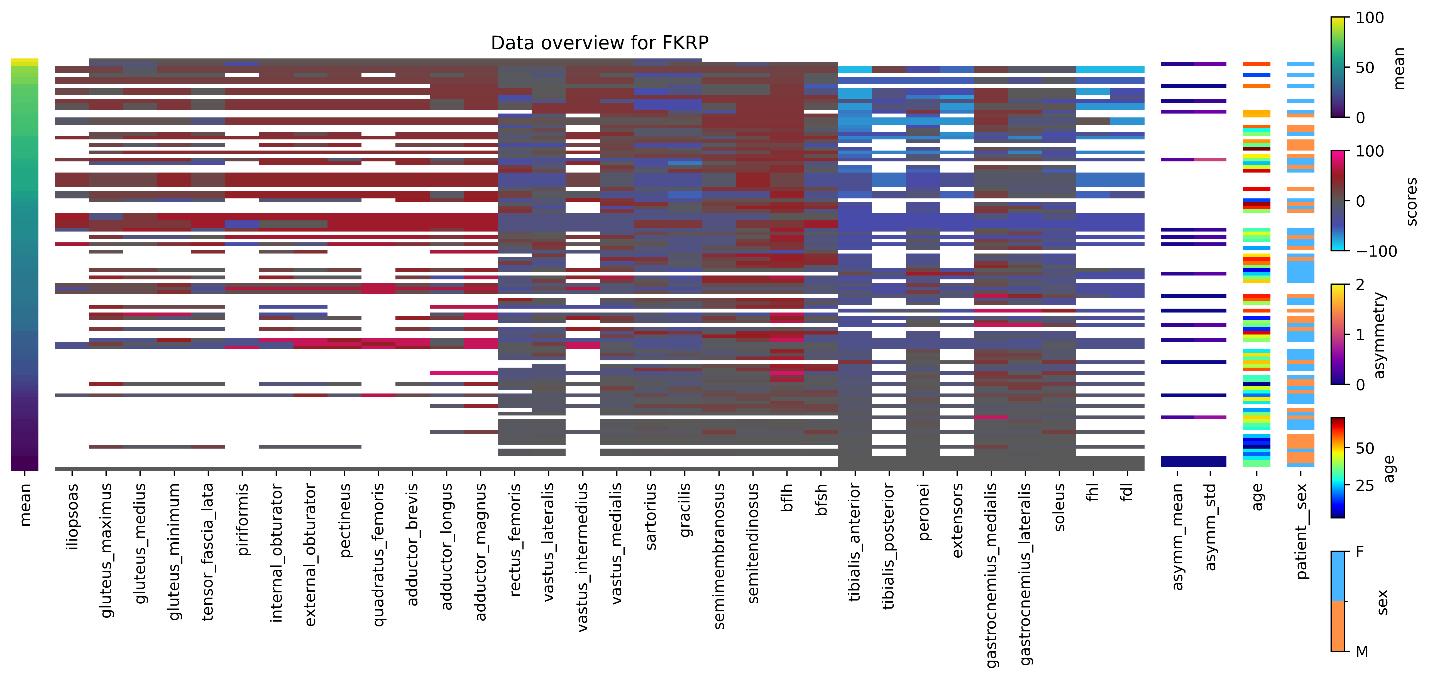
Fig. S9: Heatmap of the data for FKRP. Patient samples are represented in rows and features in columns. Rows are sorted by mean fat score, with late-stage patients in the upper rows and early-stage patients in the lower rows. Asymmetry is calculated as the difference between each left and right muscle, and the mean and standard deviation of all muscles are added as features to each patient. Muscle abbreviations: biceps femoris long head (bflh), biceps femoris short head (bfsh), flexor hallucis longus (fhl), flexor digitorum longus (fdl). The extensor digitorum longus and extensor hallucis longus have been grouped and named “extensors”.

#
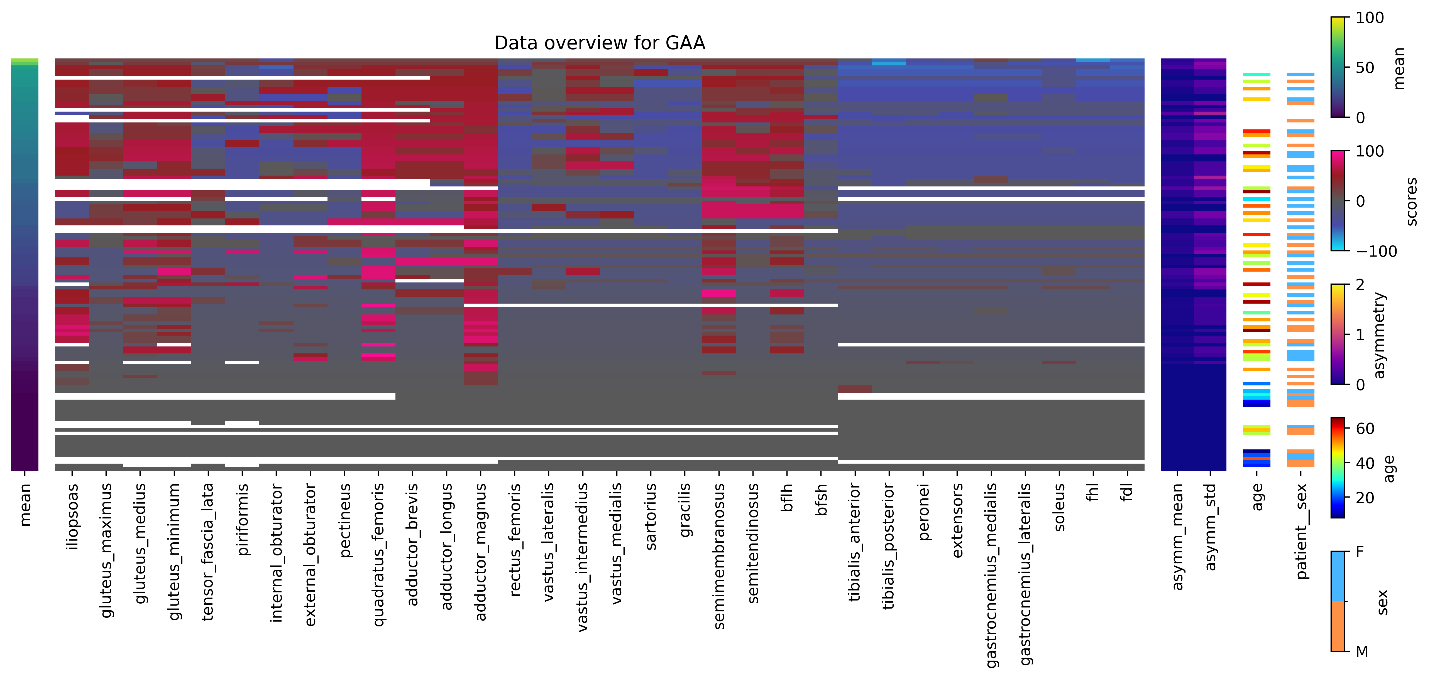
Fig. S10: Heatmap of the data for GAA. Patient samples are represented in rows and features in columns. Rows are sorted by mean fat score, with late-stage patients in the upper rows and early-stage patients in the lower rows. Asymmetry is calculated as the difference between each left and right muscle, and the mean and standard deviation of all muscles are added as features to each patient. Muscle abbreviations: biceps femoris long head (bflh), biceps femoris short head (bfsh), flexor hallucis longus (fhl), flexor digitorum longus (fdl). The extensor digitorum longus and extensor hallucis longus have been grouped and named “extensors”.

#
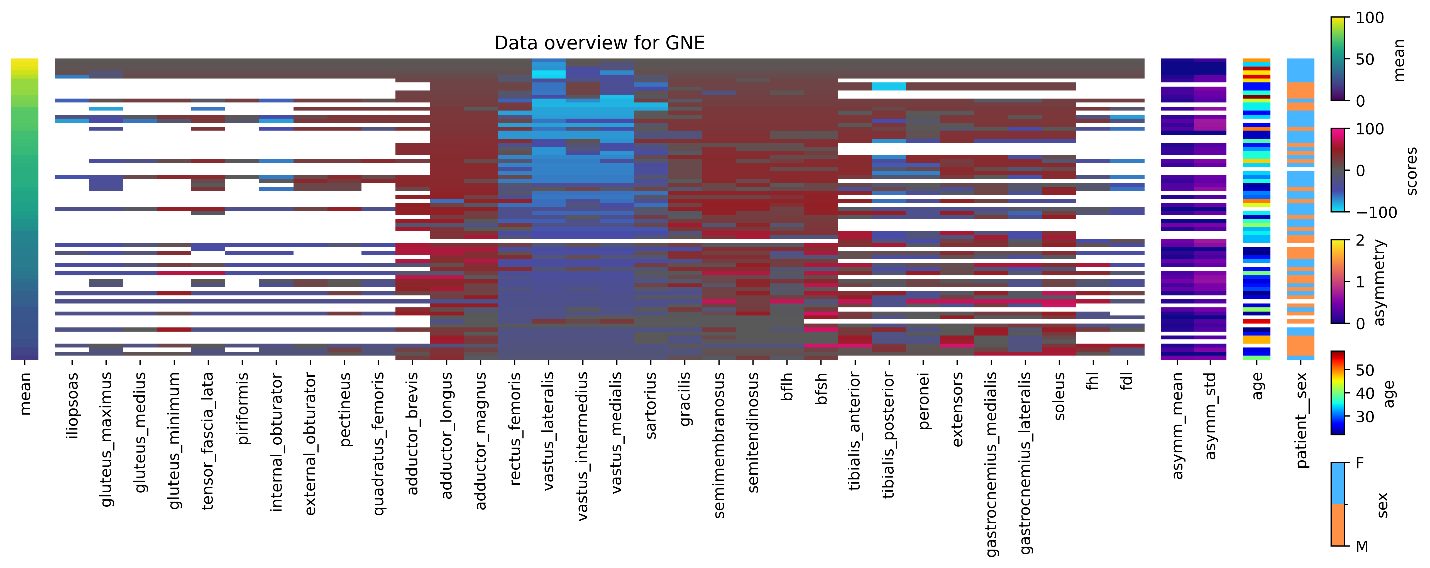
Fig. S11: Heatmap of the data for GNE. Patient samples are represented in rows and features in columns. Rows are sorted by mean fat score, with late-stage patients in the upper rows and early-stage patients in the lower rows. Asymmetry is calculated as the difference between each left and right muscle, and the mean and standard deviation of all muscles are added as features to each patient. Muscle abbreviations: biceps femoris long head (bflh), biceps femoris short head (bfsh), flexor hallucis longus (fhl), flexor digitorum longus (fdl). The extensor digitorum longus and extensor hallucis longus have been grouped and named “extensors”.

#
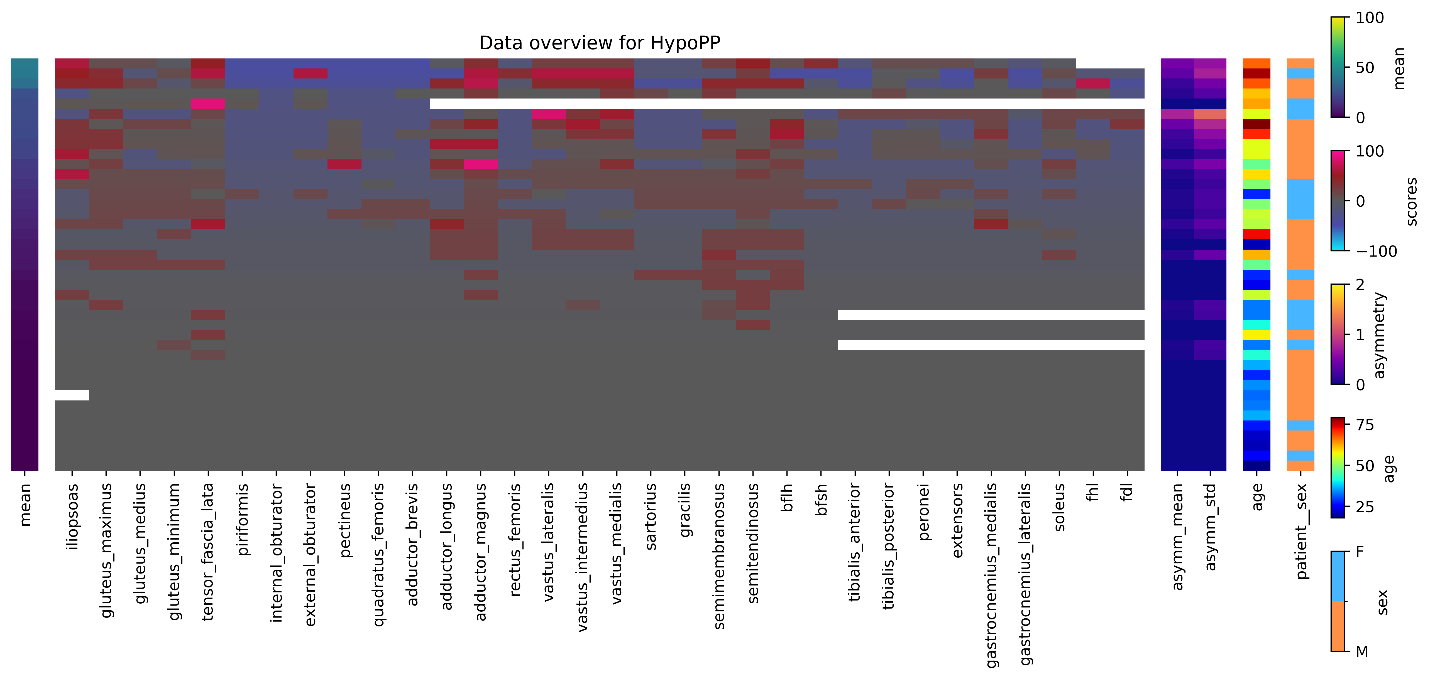
Fig. S12: Heatmap of the data for HypoPP. Patient samples are represented in rows and features in columns. Rows are sorted by mean fat score, with late-stage patients in the upper rows and early-stage patients in the lower rows. Asymmetry is calculated as the difference between each left and right muscle, and the mean and standard deviation of all muscles are added as features to each patient. Muscle abbreviations: biceps femoris long head (bflh), biceps femoris short head (bfsh), flexor hallucis longus (fhl), flexor digitorum longus (fdl). The extensor digitorum longus and extensor hallucis longus have been grouped and named “extensors”.

#
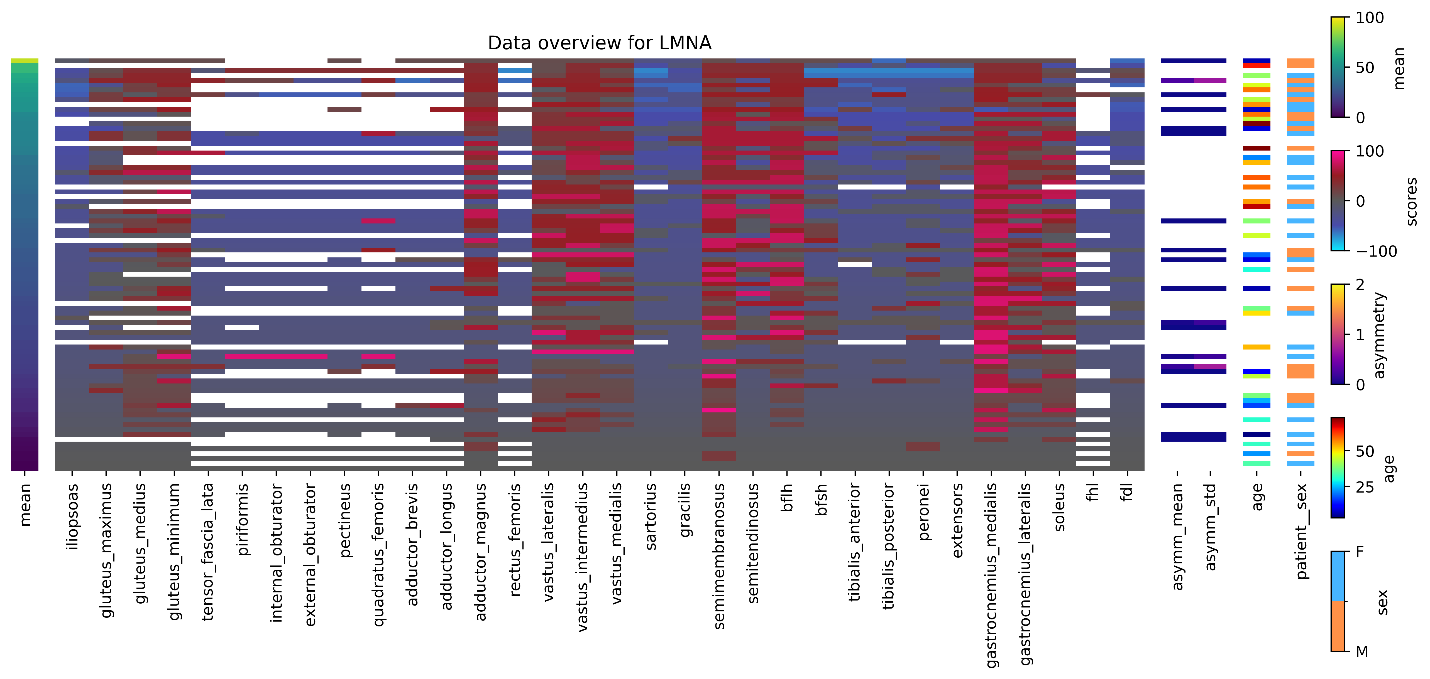
Fig. S13: Heatmap of the data for LMNA. Patient samples are represented in rows and features in columns. Rows are sorted by mean fat score, with late-stage patients in the upper rows and early-stage patients in the lower rows. Asymmetry is calculated as the difference between each left and right muscle, and the mean and standard deviation of all muscles are added as features to each patient. Muscle abbreviations: biceps femoris long head (bflh), biceps femoris short head (bfsh), flexor hallucis longus (fhl), flexor digitorum longus (fdl). The extensor digitorum longus and extensor hallucis longus have been grouped and named “extensors”.

#
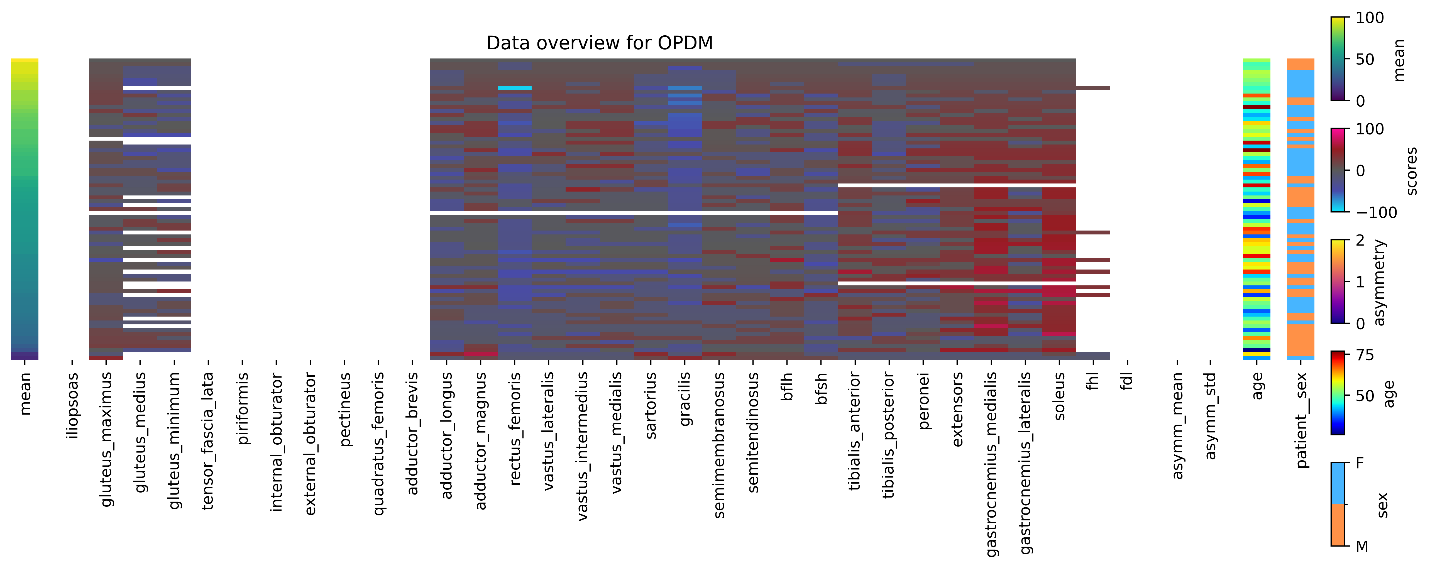
Fig. S14: Heatmap of the data for OPDM. Patient samples are represented in rows and features in columns. Rows are sorted by mean fat score, with late-stage patients in the upper rows and early-stage patients in the lower rows. Asymmetry is calculated as the difference between each left and right muscle, and the mean and standard deviation of all muscles are added as features to each patient. Muscle abbreviations: biceps femoris long head (bflh), biceps femoris short head (bfsh), flexor hallucis longus (fhl), flexor digitorum longus (fdl). The extensor digitorum longus and extensor hallucis longus have been grouped and named “extensors”.

#
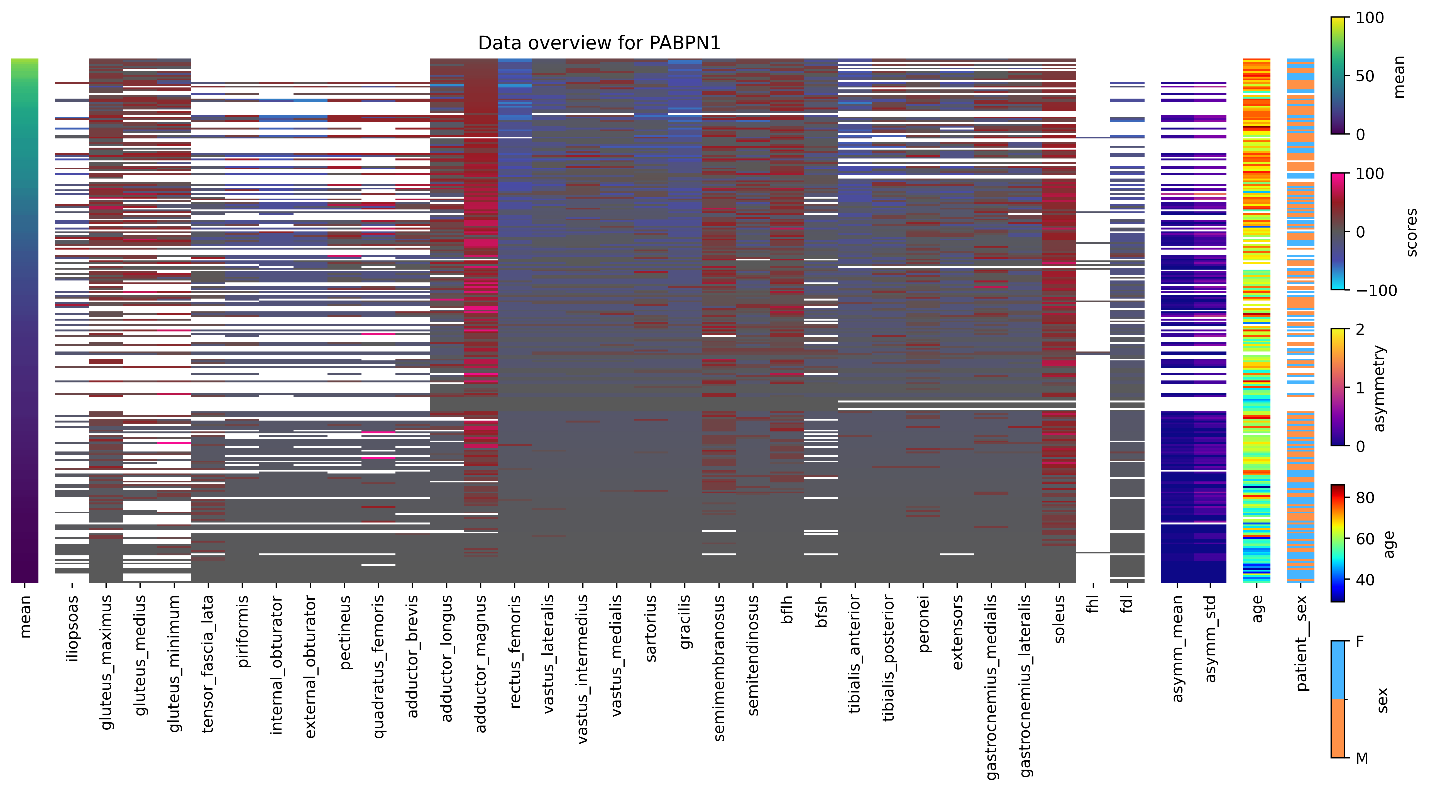
Fig. S15: Heatmap of the data for PABPN1. Patient samples are represented in rows and features in columns. Rows are sorted by mean fat score, with late-stage patients in the upper rows and early-stage patients in the lower rows. Asymmetry is calculated as the difference between each left and right muscle, and the mean and standard deviation of all muscles are added as features to each patient. Muscle abbreviations: biceps femoris long head (bflh), biceps femoris short head (bfsh), flexor hallucis longus (fhl), flexor digitorum longus (fdl). The extensor digitorum longus and extensor hallucis longus have been grouped and named “extensors”.

#
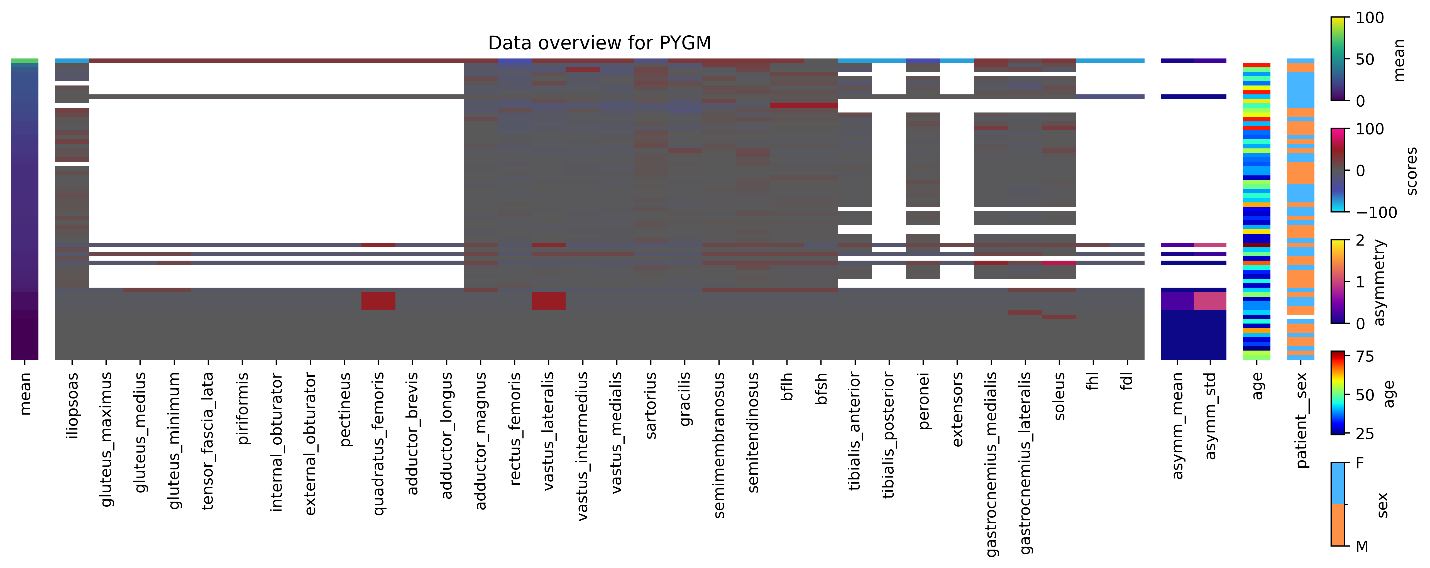
Fig. S16: Heatmap of the data for PYGM. Patient samples are represented in rows and features in columns. Rows are sorted by mean fat score, with late-stage patients in the upper rows and early-stage patients in the lower rows. Asymmetry is calculated as the difference between each left and right muscle, and the mean and standard deviation of all muscles are added as features to each patient. Muscle abbreviations: biceps femoris long head (bflh), biceps femoris short head (bfsh), flexor hallucis longus (fhl), flexor digitorum longus (fdl). The extensor digitorum longus and extensor hallucis longus have been grouped and named “extensors”.

#
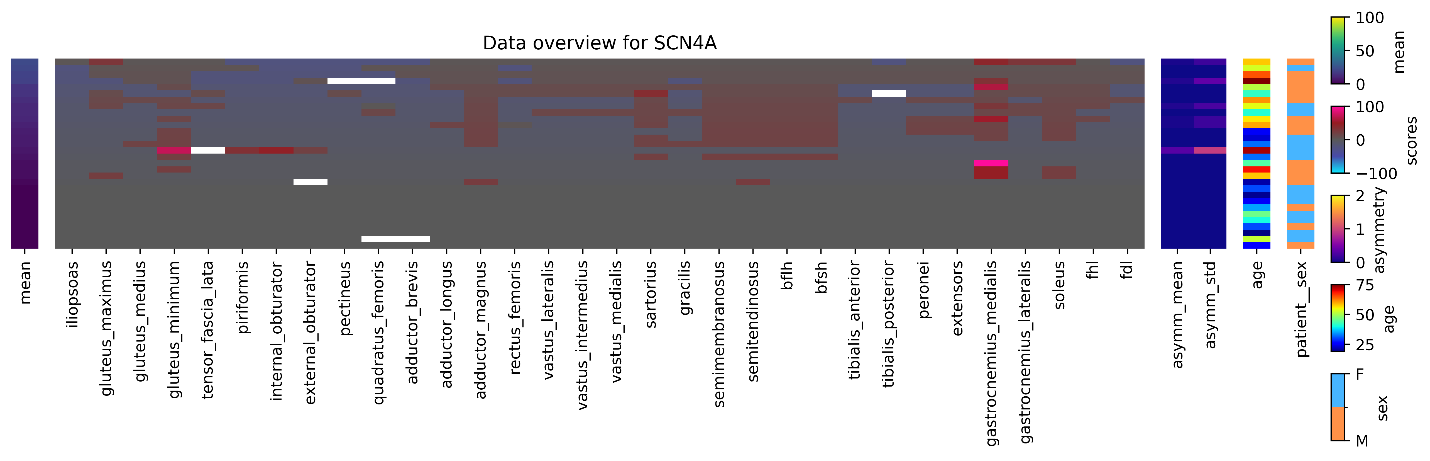
Fig. S17: Heatmap of the data for SCN4A. Patient samples are represented in rows and features in columns. Rows are sorted by mean fat score, with late-stage patients in the upper rows and early-stage patients in the lower rows. Asymmetry is calculated as the difference between each left and right muscle, and the mean and standard deviation of all muscles are added as features to each patient. Muscle abbreviations: biceps femoris long head (bflh), biceps femoris short head (bfsh), flexor hallucis longus (fhl), flexor digitorum longus (fdl). The extensor digitorum longus and extensor hallucis longus have been grouped and named “extensors”.

#
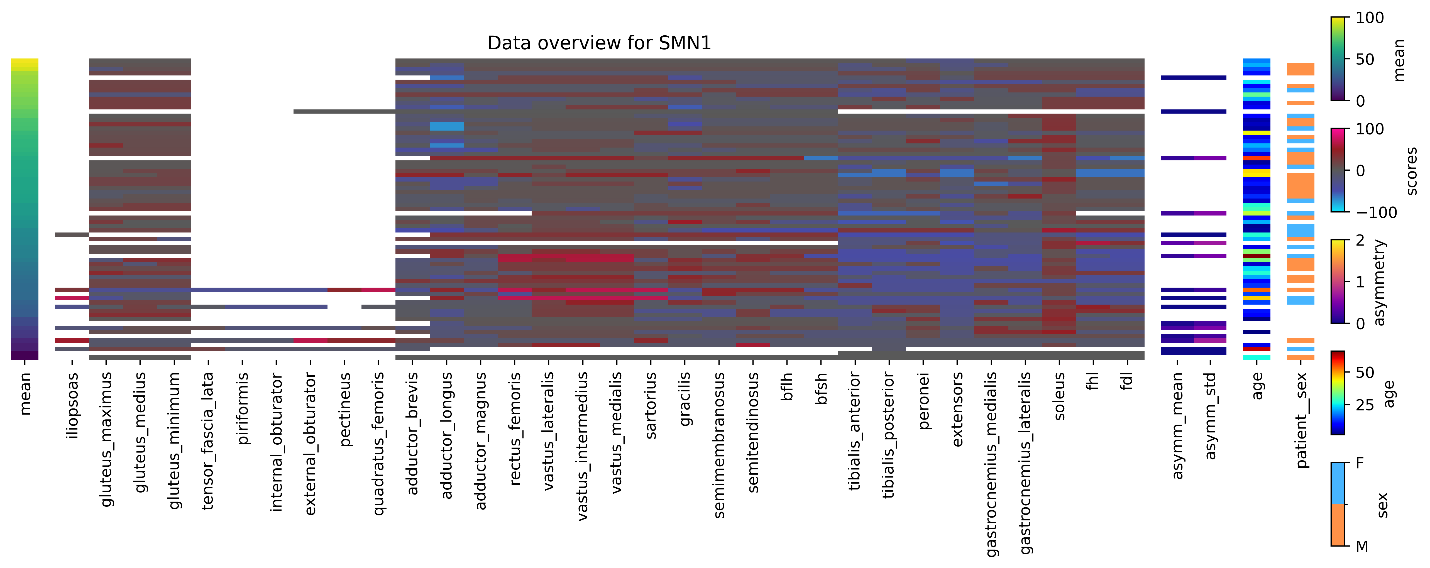
Fig. S18: Heatmap of the data for SMN1. Patient samples are represented in rows and features in columns. Rows are sorted by mean fat score, with late-stage patients in the upper rows and early-stage patients in the lower rows. Asymmetry is calculated as the difference between each left and right muscle, and the mean and standard deviation of all muscles are added as features to each patient. Muscle abbreviations: biceps femoris long head (bflh), biceps femoris short head (bfsh), flexor hallucis longus (fhl), flexor digitorum longus (fdl). The extensor digitorum longus and extensor hallucis longus have been grouped and named “extensors”.

#
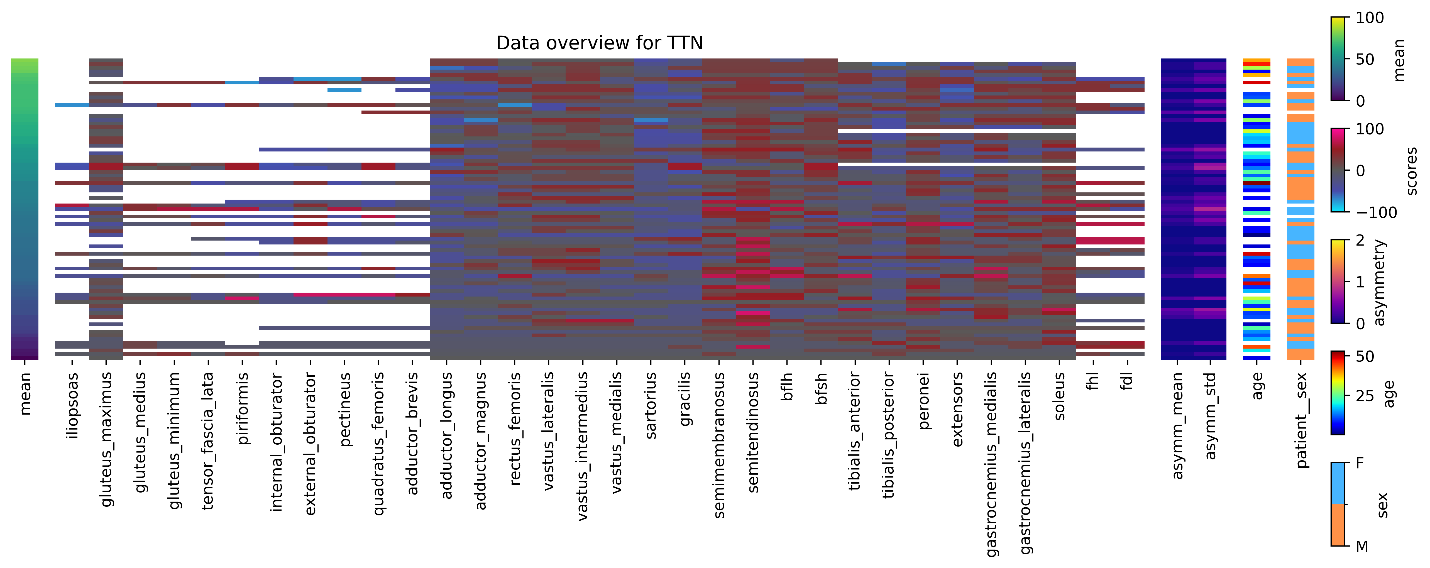
Fig. S19: Heatmap of the data for TTN. Patient samples are represented in rows and features in columns. Rows are sorted by mean fat score, with late-stage patients in the upper rows and early-stage patients in the lower rows. Asymmetry is calculated as the difference between each left and right muscle, and the mean and standard deviation of all muscles are added as features to each patient. Muscle abbreviations: biceps femoris long head (bflh), biceps femoris short head (bfsh), flexor hallucis longus (fhl), flexor digitorum longus (fdl). The extensor digitorum longus and extensor hallucis longus have been grouped and named “extensors”.

#
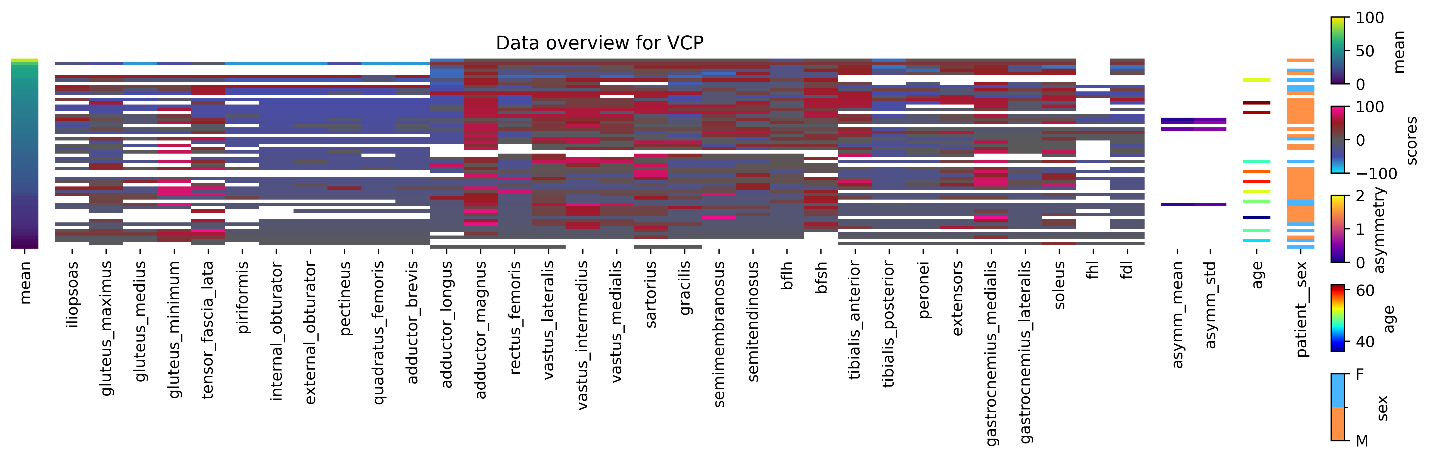
Fig. S20: Heatmap of the data for VCP. Patient samples are represented in rows and features in columns. Rows are sorted by mean fat score, with late-stage patients in the upper rows and early-stage patients in the lower rows. Asymmetry is calculated as the difference between each left and right muscle, and the mean and standard deviation of all muscles are added as features to each patient. Muscle abbreviations: biceps femoris long head (bflh), biceps femoris short head (bfsh), flexor hallucis longus (fhl), flexor digitorum longus (fdl). The extensor digitorum longus and extensor hallucis longus have been grouped and named “extensors”.


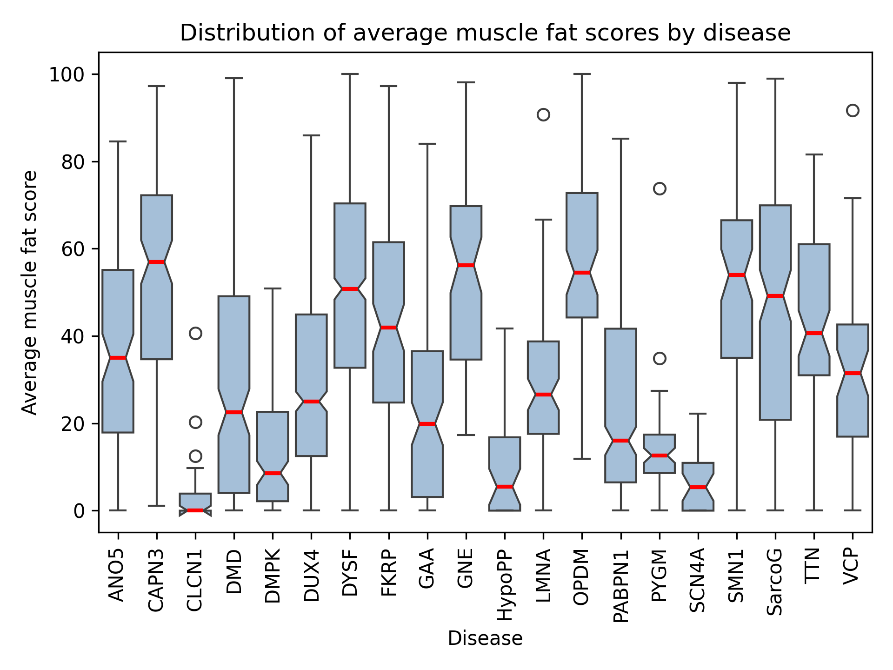


**Fig. S21**: Distribution of the average muscle fat score for each disease with median values shown in red.

| **Disease** | **AUC** | **AUPRC** | **Precision** | **Recall** | **F1-score** |
| --- | --- | --- | --- | --- | --- |
| **ANO5** | 0.91647 | 0.51185 | 0.46269 | 0.53448 | 0.49600 |
| **CAPN3** | 0.93437 | 0.64147 | 0.59477 | 0.65468 | 0.62329 |
| **CLCN1** | 0.92573 | 0.12152 | 0.12987 | 0.32258 | 0.18519 |
| **DMD** | 0.95147 | 0.71235 | 0.67045 | 0.65922 | 0.66479 |
| **DMPK** | 0.97063 | 0.81136 | 0.68966 | 0.70423 | 0.69686 |
| **DUX4** | 0.94849 | 0.86041 | 0.81223 | 0.76074 | 0.78564 |
| **DYSF** | 0.96745 | 0.90654 | 0.87810 | 0.77609 | 0.82395 |
| **FKRP** | 0.92819 | 0.64475 | 0.63725 | 0.58036 | 0.60748 |
| **GAA** | 0.95675 | 0.76402 | 0.67500 | 0.69828 | 0.68644 |
| **GNE** | 0.98346 | 0.87396 | 0.75294 | 0.85333 | 0.80000 |
| **HypoPP** | 0.92031 | 0.22655 | 0.22222 | 0.39024 | 0.28319 |
| **LMNA** | 0.96032 | 0.75044 | 0.67089 | 0.62353 | 0.64634 |
| **OPDM** | 0.97135 | 0.80089 | 0.77941 | 0.68831 | 0.73103 |
| **PABPN1** | 0.97748 | 0.91237 | 0.87868 | 0.82986 | 0.85357 |
| **PYGM** | 0.96486 | 0.73334 | 0.72581 | 0.67164 | 0.69767 |
| **SCN4A** | 0.92258 | 0.18234 | 0.16667 | 0.33333 | 0.22222 |
| **SMN1** | 0.95019 | 0.78244 | 0.79032 | 0.69014 | 0.73684 |
| **SarcoG** | 0.97720 | 0.83292 | 0.78363 | 0.78824 | 0.78592 |
| **TTN** | 0.95615 | 0.74964 | 0.69136 | 0.69136 | 0.69136 |
| **VCP** | 0.95061 | 0.59153 | 0.61017 | 0.62069 | 0.61538 |

**Supplementary Table 1:** performance of the model ensemble for each disease.

#
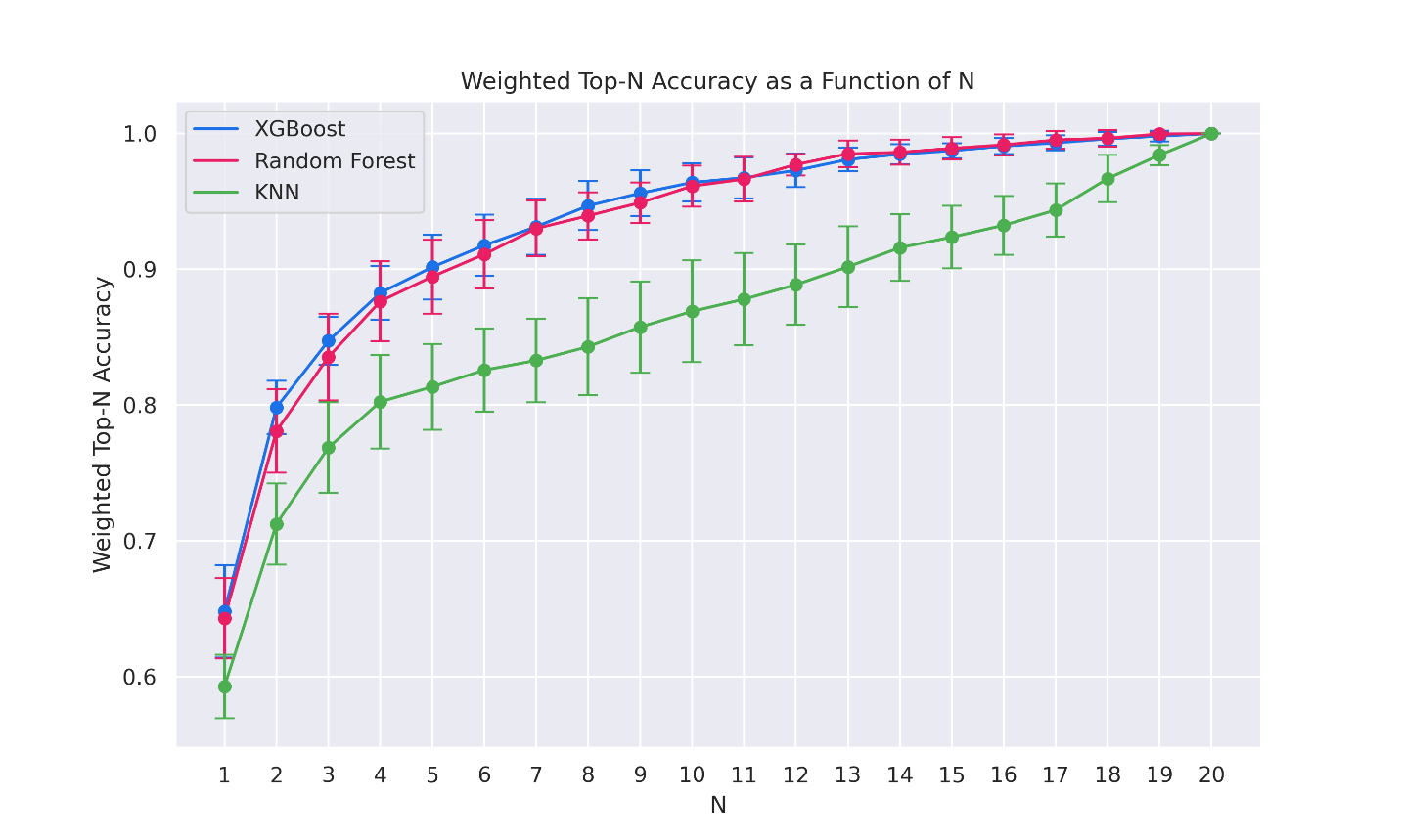


**Fig. S22**: Weighted top-N accuracy as a function of N for each classifier. When N=1, the weighted top-N accuracy is equivalent to the balanced accuracy. When N=20, the weighted top-N accuracy achieves perfection and is meaningless, as all classes are considered in the calculation of the metric.

#
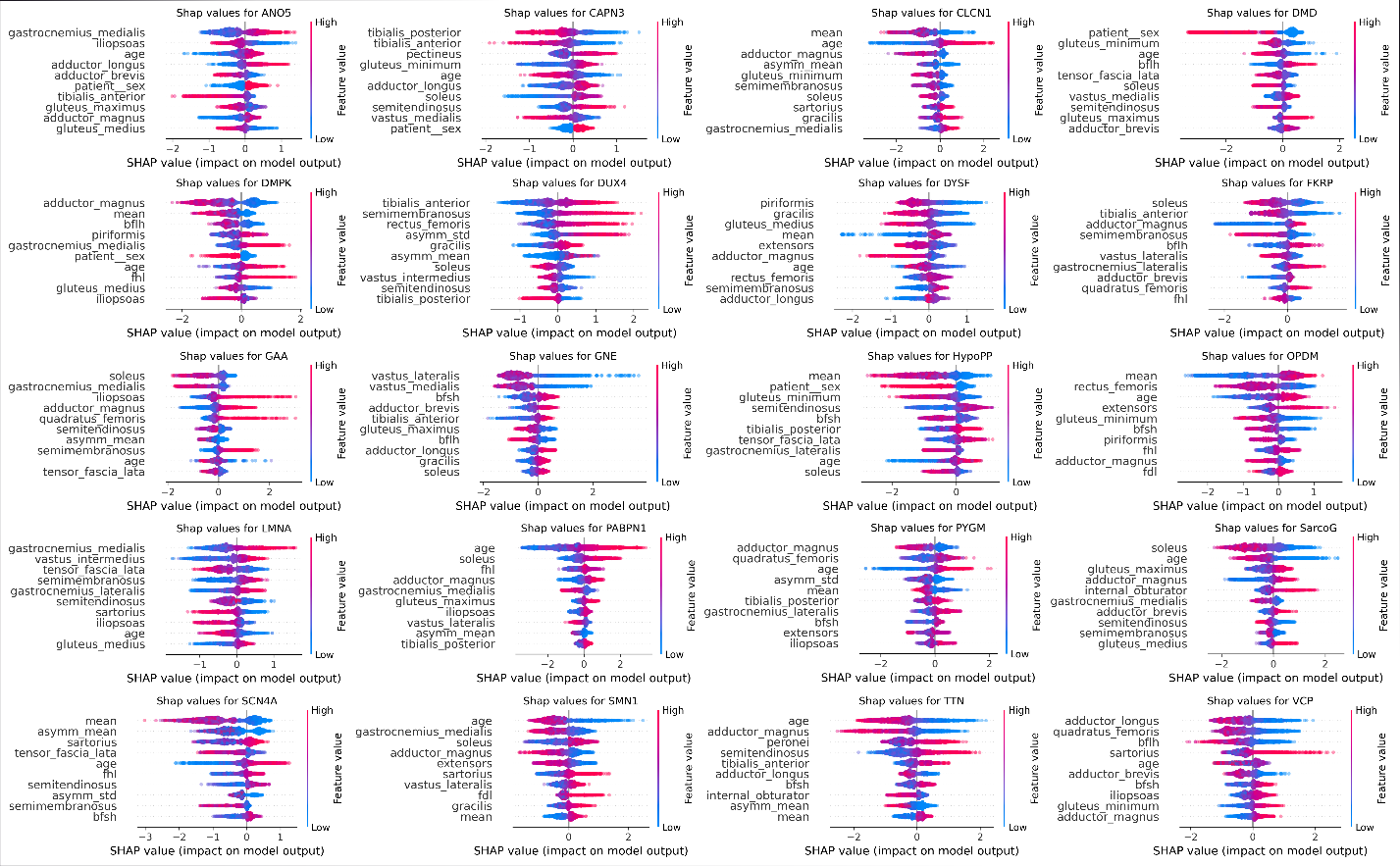


**Fig. S23:** SHAP values of the 10 most important features in predicting each disease. Each subplot corresponds to a different disease. Positive SHAP values indicate a positive impact on the prediction (increase in odds of predicting the target disease) and vice versa. Feature values are colour-coded: “high” is equivalent to the maximum feature value, and “low” is equivalent to the minimum feature value.

#
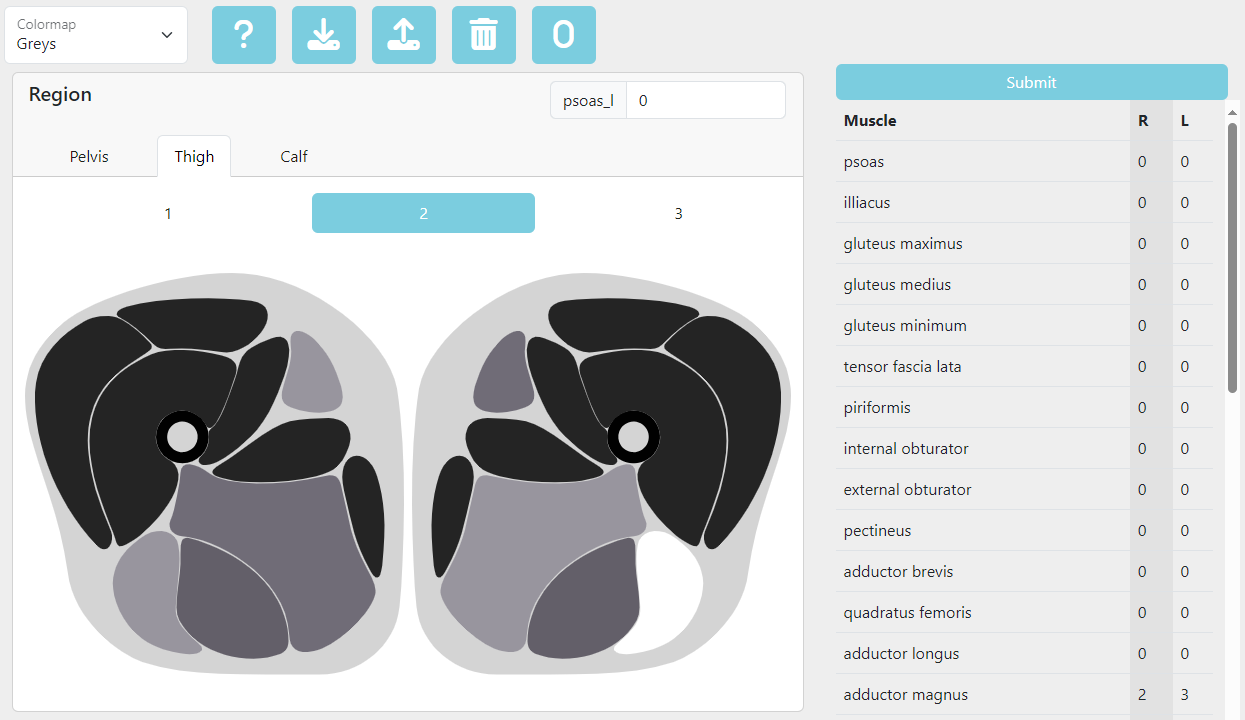


**Fig. S24**: Screenshot of the interactive user interface of the web deployment. The platform offers an interactive representation of the leg muscles, allowing the user to score each patient visually.

**Myo-Guide Consortium Members**

- **Aisha Munawar Sheikh**, Copenhagen Neuromuscular Center, Rigshospitalet, Copenhagen University Hospital, Copenhagen, Denmark
- **Alejandro Gonzalez Chamorro**, John Walton Muscular Dystrophy Research Centre, Newcastle University, Newcastle upon Tyne, United Kigndom
- **Ali Asghar Okhovat**, Neurology Department, Shariati Hospital, Neuromuscular Research Center, Tehran University of Medical Sciences, Tehran, Iran
- **Alicia Alonso-Jiménez**, Neuromuscular Reference Center. Department of Neurology. Universitair Ziekenhuis van Antwerpen. Universiteit Antwerpen. Antwerp, Belgium
- **Andre Macedo Serafim da Silva**, Department of Neurology, Faculdade de Medicina da Universidade de São Paulo (FMUSP), São Paulo, Brazil
- **Angela Berardinelli**, Child and Adolescent Neuropsychiatry Department, Mondino IRCCS Foundation, Pavia, Italy
- **Anna Lia Frongia**, Fondazione Policlinico Universitario Agostino Gemelli, Rome, Italy
- **Anna Pichiecchio**, Department of Brain and Behavioural Sciences, Unversity of Pavia, Italy; Advanced Imaging and AI Center, Mondino IRCCS Foundation, Pavia, Italy
- **Anna Sarkozy**, Dubowitz Neuromuscular Centre, UCL Great Ormond Street Institute of Child Health & Great Ormond Street Hospital, London, UK
- **Anne Marie Childs**, Leeds Teaching Hospitals NHS Trust, Leeds, United Kingdom
- **Anne-Sophie Vibæk Eisum**, Copenhagen Neuromuscular Center, Rigshospitalet, Copenhagen University Hospital, Copenhagen, Denmark
- **Atchayaram Nalini**, National Institute of Mental Health and Neurosciences (NIMHANS), Bengaluru, India
- **Benjamín Pizarro-Galleguillos**, Programa de Doctorado en Ciencias Médicas y Especialidad, Escuela de Postgrado Facultad de Medicina Universidad de Chile, Santiago, Chile
- **Bianca Buchignani** ,Department of Translational Research and of New Surgical and Medical Technologies, University of Pisa, Pisa, Italy
- **Carla Bolaño-Díaz**, John Walton Muscular Dystrophy Research Centre, Newcastle University, Newcastle upon Tyne, United Kigndom
- **Chiara Marini Bettolo**, John Walton Muscular Dystrophy Research Centre, Newcastle University, Newcastle upon Tyne, United Kigndom
- **Chongbo Zhao**, Department of Neurology, Huashan Hospital, Fudan University, Shanghai, China
- **Claudia Brogna**, Fondazione Policlinico Universitario Agostino Gemelli, Rome, Italy
- **Claudia Nuñez-Peralta**, Musculoskeletal service, Department of Radiology, Hospital de la Santa Creu i Sant Pau, Barcelona, Spain
- **Cristina Domínguez-González**, Neuromuscular Disorders Unit, Neurology Department, Hospital 12 de Octubre, Madrid, Spain
- **Cristina Martos-Lozano**, Leeds Teaching Hospitals NHS Trust, Leeds, United Kingdom
- **Darryl Darian Suryadi**, John Walton Muscular Dystrophy Research Centre, Newcastle University, Newcastle upon Tyne, United Kigndom
- **David Bendahan**, Aix-Marseille University, CRMBM, CNRS UMR 7339, Marseille, France
- **David Gomez Andres**, Hospital Universitari Vall d'Hebron, Barcelona, Spain
- **Debora Mucida Alvim**, John Walton Muscular Dystrophy Research Centre, Newcastle University, Newcastle upon Tyne, United Kigndom
- **Edmar Zanoteli**, Department of Neurology, Faculdade de Medicina da Universidade de São Paulo (FMUSP), São Paulo, Brazil
- **Edoardo Malfatti**, Paris Est University, APHP Henri-Mondor University Hospital, Créteil, France
- **Elena Stebbings**, John Walton Muscular Dystrophy Research Centre, Newcastle University, Newcastle upon Tyne, United Kigndom
- **Elisa De La Cruz**, Centre de référence des maladies du motoneurone, department of neurology, Montpellier University Hospital, Montpellier, France
- **Emma Matthews**, St George's University and St George's University Hospitals NHS Foundation Trust, London, United Kingdom
- **Emmanuelle Le Bars**, Department of Neuroradiology, I2FH platform, Montpellier University Hospital, Montpellier, France
- **Erik H Niks**, Department of Neurology, Leiden University Medical Center, Leiden, The Netherlands
- **Eugenio Mercuri**, Pediatric Neurology, Department of Woman and Child Health and Public Health, Child Health Area, Università Cattolica del Sacro Cuore, Rome, Italy
- **Filipe Tupinamba Di Pace**, Department of Neurology, Faculdade de Medicina da Universidade de São Paulo (FMUSP), São Paulo, Brazil
- **Florence Esselin**, Centre de référence des maladies du motoneurone, department of neurology, Montpellier University Hospital, Montpellier, France
- **Gabriel Aguilera**, Hospital Clínico Universidad de Chile, Santiago de Chile, Chile
- **Gema Iglesias Escalera**, Hospital Puerta de Hierro, Madrid, Spain
- **Giorgio Tasca**, John Walton Muscular Dystrophy Research Centre, Newcastle University, Newcastle upon Tyne, United Kigndom
- **Giovanni Baranello**, Dubowitz Neuromuscular Centre, UCL Great Ormond Street Institute of Child Health & Great Ormond Street Hospital, London, UK
- **Goknur Selen Kocak** , John Walton Muscular Dystrophy Research Centre, Newcastle University, Newcastle upon Tyne, United Kigndom
- **Grete Andersen**, Copenhagen Neuromuscular Center, Rigshospitalet, Copenhagen University Hospital, Copenhagen, Denmark
- **Guja Astrea**, Department of Neuroscience, IRCCS Stella Maris Foundation, 56128 Pisa, Italy
- **Hermien E Kan**, C.J. Gorter MRI Center, Department of Radiology, Leiden University Medical Center, Leiden, The Netherlands
- **Holly Borland**, John Walton Muscular Dystrophy Research Centre, Newcastle University, Newcastle upon Tyne, United Kigndom
- **Huahua Zhong**, Department of Neurology, Huashan Hospital, Fudan University, Shanghai, China
- **Ian C. Smith**, Ottawa Hospital Research Institute, Ottawa, Canada
- **Ian Wilson**, Magnetic Resonance Centre, Newcastle University, Newcastle upon Tyne, United Kingdom
- **James Lilleker**, Northern Care Alliance NHS Foundation Trust, Manchester, United Kingdom
- **Jaume Bacardit**, Interdisciplinary Computing and Complex BioSystems research group, School of Computing, Newcastle University, Newcastle upon Tyne, UK
- **Javier Sotoca**, Neuromuscular Disorders Unit, Neurology Department, Hospital Universitari Vall d'Hebron, Barcelona, Spain
- **Jeannette Kraft**, Leeds Teaching Hospitals NHS Trust, Leeds, United Kingdom
- **Jinhong Shin**, Department of Neurology, Pusan National University School of Medicine, Busan, Republic of Korea
- **Jodi Warman-Chardon**, Department of Medicine (Neurology), The Ottawa Hospital, Ottawa, Canada; Genetics, Children’s Hospital of Eastern Ontario, Ottawa, Canada
- **John Vissing**, Copenhagen Neuromuscular Center, Rigshospitalet, Copenhagen University Hospital, Copenhagen, Denmark
- **Jonas Jalili Pedersen**, Copenhagen Neuromuscular Center, Rigshospitalet, Copenhagen University Hospital, Copenhagen, Denmark
- **Jong-Mok Lee**, Department of Neurology, Kyungpook National University, Kyungpook National University Hospital, Daegu, South Korea
- **Jordi Díaz-Manera**, John Walton Muscular Dystrophy Research Centre, Newcastle University, Newcastle upon Tyne, United Kigndom
- **Jorge Alonso-Pérez**, Neuromuscular Disease Unit, Neurology Department, Hospital Universitario Nuestra Señora de Candelaria, Tenerife, Spain.
- **Jorge Alonso-Pérez**, Neuromuscular Disease Unit, Neurology Department, Hospital Universitario Nuestra Señora de Candelaria, Tenerife, Spain.
- **Jorge Bevilacqua Rivas**, Hospital Clínico Universidad de Chile, Santiago de Chile, Chile
- **Jorge Díaz-Jara**, Hospital Clínico Universidad de Chile, Santiago de Chile, Chile
- **Jose Verdu-Diaz**, John Walton Muscular Dystrophy Research Centre, Newcastle University, Newcastle upon Tyne, United Kigndom
- **Julia Dahlqvist**, Copenhagen Neuromuscular Center, Rigshospitalet, Copenhagen University Hospital, Copenhagen, Denmark
- **Karen Pysden**, Leeds Teaching Hospitals NHS Trust, Leeds, United Kingdom
- **Kieren Hollingsworth**, Translational and Clinical Research Institute, Newcastle University, Newcastle upon Tyne, United Kingdom
- **Kiran Polavarapu**, Children's Hospital of Eastern Ontario Research Institute, Ottawa, Canada.
- **Lara Cristiano**, Fondazione Policlinico Universitario Agostino Gemelli, Rome, Italy
- **Laura Bermejo-Guerrero**, Neuromuscular Disorders Unit, Neurology Department, Hospital 12 de Octubre, Madrid, Spain
- **Laura Fionda** , Neuromuscular and Rare Disease Centre, Neurology Unit, Sant’Andrea Hospital, Rome, Italy
- **Laura Nørager Jacobsen**, Copenhagen Neuromuscular Center, Rigshospitalet, Copenhagen University Hospital, Copenhagen, Denmark
- **Laura Tufano**, Department of Neuroscience, Mental Health and Sensory Organs (NESMOS), SAPIENZA University of Rome, Rome, Italy
- **Luke Perry**, Dubowitz Neuromuscular Centre, UCL Great Ormond Street Institute of Child Health & Great Ormond Street Hospital, London, UK
- **Marcelo Rugiero**, Hospital Italiano de Buenos Aires, Buenos Aires, Argentina
- **Mariela Bettini**, Hospital Italiano de Buenos Aires, Buenos Aires, Argentina
- **Mark Roberts**, Northern Care Alliance NHS Foundation Trust, Manchester, United Kingdom
- **Matteo Garibaldi**, Department of Neuroscience, Mental Health and Sensory Organs (NESMOS), SAPIENZA University of Rome, Rome, Italy
- **Mauro Monforte**, UOC di Neurologia, Fondazione Policlinico Universitario Agostino Gemelli IRCCS, Rome, Italy
- **Melissa Hooijmans**, Department of Radiology and Nuclear Medicine, Amsterdam UMC, University of Amsterdam, Amsterdam Movement Sciences, Amsterdam, The Netherlands
- **Michela Guglieri**, John Walton Muscular Dystrophy Research Centre, Newcastle University, Newcastle upon Tyne, United Kigndom
- **Montse Olivé** , Neuromuscular Disorders Unit, Department of Neurology, Hospital de la Santa Creu i Sant Pau, and Biomedical Research Institute Sant Pau (IIB Sant Pau). Barcelona, Spain; Centro de Investigaciones Biomédicas en Red en Enfermedades Raras (CIBERER), Madrid, Spain
- **Nanna Scharff Poulsen**, Copenhagen Neuromuscular Center, Rigshospitalet, Copenhagen University Hospital, Copenhagen, Denmark
- **Nicoline Løkken**, Copenhagen Neuromuscular Center, Rigshospitalet, Copenhagen University Hospital, Copenhagen, Denmark
- **Peter Krkoska**, University Hospital Brno, Brno, Czech Republic
- **Ricard Rojas-García** , Neuromuscular Disorders Unit, Department of Neurology, Hospital de la Santa Creu i Sant Pau, and Biomedical Research Institute Sant Pau (IIB Sant Pau). Barcelona, Spain
- **Robert Carlier**, University Hospital Raymond-Poincaré, Garches, France
- **Roberta Battini**, Department of Clinical and Experimental Medicine, University of Pisa, 56126 Pisa, Italy
- **Rocco Constanzo**, Department of Neuroscience, Mental Health and Sensory Organs (NESMOS), SAPIENZA University of Rome, Rome, Italy
- **Rosa Pasquariello**, Department of Neuroscience, IRCCS Stella Maris Foundation, 56128 Pisa, Italy
- **Sam Fitzsimmons**, John Walton Muscular Dystrophy Research Centre, Newcastle University, Newcastle upon Tyne, United Kigndom
- **Sara Bortolani**, UOC di Neurologia, Fondazione Policlinico Universitario Agostino Gemelli IRCCS, Rome, Italy
- **Seena Vengalil**, National Institute of Mental Health and Neurosciences (NIMHANS), Bengaluru, India
- **Shahram Attarian**, Reference center for neuromuscular disorders CHU La Timone, Aix-Marseille University, Marseille, France
- **Shahriar Nafissi**, Neurology Department, Shariati Hospital, Neuromuscular Research Center, Tehran University of Medical Sciences, Tehran, Iran
- **Shona Haston** , John Walton Muscular Dystrophy Research Centre, Newcastle University, Newcastle upon Tyne, United Kigndom
- **Silvia Nicolosi**, University of Pavia, Pavia, Italy; Mondino IRCCS Foundation, Pavia, Italy
- **Sniya Sudhakar**, Department of Neuroradiology Great Ormond Street Hospital for Children NHS Foundation Trust, London, UK
- **Sonja Desirée Holm-Yildiz**, Copenhagen Neuromuscular Center, Rigshospitalet, Copenhagen University Hospital, Copenhagen, Denmark
- **Sravan Kumar Reddy Edamakanti** , National Institute of Mental Health and Neurosciences (NIMHANS), Bengaluru, India
- **Sushan Luo**, Department of Neurology, Huashan Hospital, Fudan University, Shanghai, China
- **Thierry Chaptal**, Montpellier University Hospital, Montpellier, France
- **Tommaso Verdolotti**, Fondazione Policlinico Universitario Agostino Gemelli, Rome, Italy
- **Vidya Nittur**, National Institute of Mental Health and Neurosciences (NIMHANS), Bengaluru, India
- **Volker Straub**, John Walton Muscular Dystrophy Research Centre, Newcastle University, Newcastle upon Tyne, United Kigndom
- **Young-Eun Park**, Department of Neurology, Pusan National University School of Medicine, Busan, Republic of Korea
