# Supplementary material for: Myo‐Guide: A Machine Learning‐Based Web Application for Neuromuscular Disease Diagnosis With MRI
Source: J Cachexia Sarcopenia Muscle. 2025 Apr 24;16(3):e13815. doi: 10.1002/jcsm.13815 (PMC12022233; doi:10.1002/jcsm.13815)
Supplement: Supplementary file 2 — Data S1 Supporting Information. [file JCSM-16-e13815-s002.docx]

**Supplementary File 2**

**SHAP and its application to model interpretation**

**Introduction to SHAP (SHapley Additive exPlanations)**

SHAP (SHapley Additive exPlanations) is a unified framework for interpreting predictions made by machine learning models. It is based on the concept of Shapley values from cooperative game theory, which were originally developed to fairly allocate payouts among players in a coalition. SHAP adapts this concept to the context of machine learning, attributing the contribution of each feature to a specific prediction in a principled and consistent manner.

SHAP values explain individual predictions, making it easier to analyse specific cases. By analysing the individual predictions of all samples used for training, we are able to obtain global insights about the model decision-making process and, therefore, about the underlying patterns of muscle involvement useful for diagnosis.

**SHAP Values**

SHAP values quantify the contribution of each feature to the difference between the model’s output for a specific sample and the mean model output across the dataset. Formally, the SHAP value for a feature is calculated as the weighted average of its marginal contributions across all possible coalitions of features.

A SHAP value is calculated for each feature of each sample for each target diagnosis; indicating the impact each feature has on the predicted probability of each diagnosis, for each sample. In our study, we trained the model with 2961 samples, 37 features and 20 possible diagnoses; resulting in a [2961x37x20] matrix of SHAP values.

Positive SHAP values indicate an increase on the model output (predicted probability of the given disease), while negative values indicate a decrease. A value close to 0 indicates low impact on the model output. For example, if a sample’s vastus medialis SHAP value for GNE is high, this indicates that the amount of intramuscular fat of the vastus medialis (whether high or low) for this sample in specific increases the predicted probability of GNE.

**SHAP Interaction Values**

While SHAP values capture the individual contribution of features, SHAP interaction values extend this by quantifying how pairs of features interact to influence the prediction. SHAP interaction values help uncover dependencies and synergistic effects between features, providing a deeper understanding of the model’s behaviour. SHAP interaction values are calculated for each sample, for each pair of features and for each disease. In our study, the interaction value matrix had dimensions [2961x37x37x20].
